# Risk of and Mortality After Acute Kidney Injury Following Cancer Treatment: A Cohort Study

**DOI:** 10.1002/cam4.70646

**Published:** 2025-02-10

**Authors:** Philip Vestergaard Munch, Mette Nørgaard, Simon Kok Jensen, Henrik Birn, Henrik Schmidt, Christian Fynbo Christiansen

**Affiliations:** ^1^ Department of Clinical Epidemiology Aarhus University Hospital Aarhus Denmark; ^2^ Department of Clinical Medicine Aarhus University Aarhus Denmark; ^3^ Department of Renal Medicine Aarhus University Hospital Aarhus Denmark; ^4^ Department of Biomedicine Aarhus University Aarhus Denmark; ^5^ Department of Oncology Aarhus University Hospital Aarhus Denmark

**Keywords:** acute kidney injury, cancer treatment, epidemiology, mortality

## Abstract

**Background:**

Acute kidney injury (AKI) can be a severe complication in cancer patients. However, uncertainty remains regarding the risk of and prognosis after AKI following cancer treatments. We therefore aimed to examine the risk of and mortality after AKI following a wide range of specific cancer treatments, including surgical procedures, anticancer drugs, and hematopoietic stem cell transplantations (HSCTs).

**Methods:**

We conducted a nationwide population‐based cohort study. We included adult patients receiving cancer treatment in Denmark from 2010 to 2024. We calculated the risk of AKI within 7 days after surgeries, 1 year after initiation of anticancer drugs, and 100 days after HSCTs. Furthermore, we examined the 1‐year mortality in patients with and without AKI following cancer treatment.

**Results:**

We identified 357,870 cancer patients. The 7‐day risk of AKI after surgery ranged from 0.3% (breast cancer surgery) to 68.9% (radical nephrectomy in kidney cancer) while the 1‐year risk following anticancer drug treatment ranged from 3.5% (cyclophosphamide in breast cancer) to 79.3% (all drugs in acute lymphatic leukemia). The 100‐day AKI risk following HSCT ranged from 20.7% (multiple myeloma) to 81.8% (leukemia). For most treatments, AKI was associated with a higher 1‐year hazard ratio and risk of death, with exceptions including radical nephrectomy in kidney cancer.

**Conclusion:**

In conclusion, several cancer treatments were associated with a high risk of AKI, and AKI was associated with increased mortality in most treatments. These findings highlight the prognostic value of assessing kidney function following specific cancer treatments in clinical practice.

**Abbreviations**
ABVD: adriamycin + bleomycin + vinblastine + dacarbazine
ADQI: Acute Disease Quality Initiative
AKI: acute kidney injury
ALL: acute lymphatic leukemia
AML: acute myeloid leukemia
BEACOPP: bleomycin + etoposide + adriamycin + cyclophosphamide + vincristine + procarbazine + prednisone
CCI: Charlson Comorbidity Index
CDK4/6i: Cyclin‐dependent kinase 4/6 inhibitors
chemo: chemotherapy
CHOP/CHOEP: cyclophosphamide + hydroxydaunorubicin + vincristine + (etoposide) + prednisone
CI: confidence interval
CLL: chronic lymphatic leukemia
CML: chronic myeloid leukemia
cyclophos: cyclophosphamide
dabra/tram: dabrafenib/trametinib
eGFR: estimated glomerular filtration rate
enco/bini: encorafenib/binimetinib
ex: excision
gen: generation
HIPEC: hyperthermic intraperitoneal chemotherapy
HR: hazard ratio
HSCT: hematopoietic stem cell transplantation
ICI: immune checkpoint inhibitors
KDIGO: Kidney Disease: Improving Global Outcomes
PARPi: poly(ADP‐ribose) polymerase inhibitors
Q1: first quartile
Q3: third quartile
res: resection
TKI: tyrosine kinase inhibitors
TURB: transurethral resection of bladder

## 1 Introduction

Over the last decades, emerging medical and surgical treatments have radically changed cancer care [1]. Despite the evident survival benefits, cancer patients often experience complications related to these new treatments [2, 3]. One potentially severe complication is acute kidney injury (AKI), which occurs in approximately 20% of cancer patients within the first year after diagnosis [4, 5, 6, 7]. Although the awareness of AKI following cancer treatments is evolving [6], the risk of AKI and especially the mortality after AKI is unknown for a wide range of cancer treatments. Most of the existing studies examining AKI following specific cancer treatments were conducted in a single center or healthcare system [8, 9, 10, 11, 12, 13, 14, 15, 16, 17, 18, 19, 20, 21, 22, 23, 24, 25, 26, 27, 28, 29]. This may have led to an underestimation of the absolute risk due to missing registration of AKI events occurring at other centers. Moreover, differences in the threshold values and time frame in the definition of AKI complicate the comparison of estimates between the studies. A previous population‐based cohort study reported the risk of AKI in patients receiving systemic cancer treatment by cancer type. However, new treatments have emerged since the end of the study period in 2014 [30]. Therefore, we conducted a Danish population‐based cohort study with complete follow‐up until February 2024 and with the Kidney Disease: Improving Global Outcomes (KDIGO) definition of AKI to estimate the absolute risks of AKI in patients with cancer, including solid tumors and hematologic malignancies, both following cancer surgery, anticancer drug treatment, and hematopoietic stem cell transplantation (HSCT); and to investigate the mortality after AKI. Knowledge about the incidence and prognostic importance of AKI after cancer treatment is important for clinical practice as it may identify cancer patients requiring additional medical attention and inform both physicians and patients about the prognosis.

## 2 Methods

### 2.1 Setting and Study Design

We designed a population‐based cohort study using data from the nationwide Danish registries covering 5.8 million residents. Denmark's National Health Service provides tax‐supported healthcare to the Danish population, ensuring free access to general practitioners and public hospitals. A unique personal identifier is assigned to all Danish residents, enabling unambiguous individual linkage among Danish registries [31], including the Danish National Patient Registry (patient registry) [32], laboratory databases [33, 34], the Danish Civil Registration System (CRS) [35], and the Danish National Pathology Registry (pathology registry) [36].

### 2.2 Study Population

**FIGURE 1:**
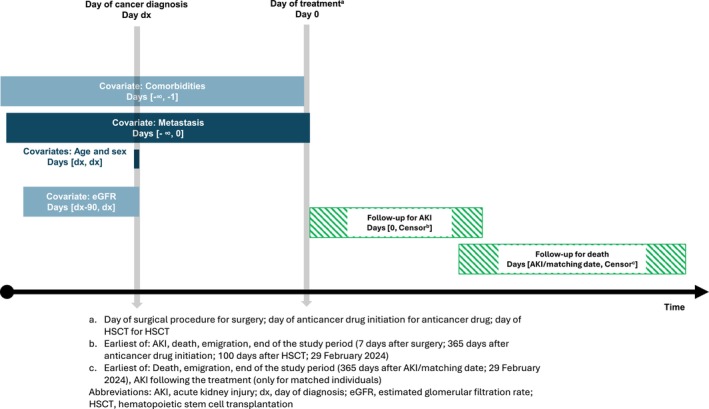
Visualization of covariate assessment and follow‐up (adapted from Schneeweiss et al. [37]).

We included adult patients (age ≥ 18 years) with incident cancer in the period January 1, 2010 to February 29, 2024. This was defined as patients with a first hospital‐recorded cancer diagnosis (solid tumors as well as hematologic malignancies) registered in the patient registry during this period and no registered cancer (except non‐melanoma skin cancer) in the period 1994–2009 (see Table S1 in Supplement for codes). The patient registry contains administrative and clinical data, including information on diagnoses, surgeries, anticancer drug treatments, and HSCT [32]. In patients with more than one cancer type, only the first recorded cancer in this period was included for each patient. Data on plasma creatinine measurements were retrieved from the Danish laboratory databases, covering the inpatient, emergency room, outpatient clinic, and general practice settings [33, 34]. We calculated the estimated glomerular filtration rate (eGFR) based on plasma creatinine measurements using the 2009 Chronic Kidney Disease Epidemiology Collaboration equation assuming non‐Black race. Baseline eGFR was defined as the median of eGFR values recorded within 90 days before the cancer diagnosis. We excluded patients without at least one creatinine measurement within 1 year before the cancer diagnosis to ensure that patients resided in areas covered by the laboratory databases. When analyzing specific treatment, we excluded patients with ongoing AKI at the start of treatment (see below), patients with one or more outpatient eGFR < 15 mL/min/1.73 m^2^, and patients with a procedure code of chronic dialysis registered before the start of treatment to increase the specificity of the AKI diagnosis. We followed all individuals from the date of first cancer diagnosis until the occurrence of death, emigration, or the end of the study period (29 February 2024; see Figure 1 for visualization of the follow‐up).

### 2.3 Treatment

We retrieved data on a wide range of cancer treatments from the patient registry, including surgeries, anticancer drug treatments, and HSCT (see Table 2, 3, 4 for the included treatments). We included information about treatment dates and treatment types using treatment codes (see Table S1 for codes).

### 2.4 Acute Kidney Injury

In alignment with guidelines from KDIGO, we defined AKI as any of the following: (1) an at least 26.5 μmol/L increase in creatinine within 48 h, (2) an at least 1.5‐fold increase in creatinine within 7 days, or (3) an at least 1.5‐fold increase in creatinine compared with the most recent creatinine measurement within the preceding 8–365 days [38]. We also identified the highest AKI stage obtained during the AKI episode (see Box 1 for the KDIGO creatinine staging of AKI). Furthermore, we determined the duration of the AKI episode defined as the period from the first measurement fulfilling the AKI criteria to the last measurement not returned to the sub‐AKI level. The duration was categorized as transient (duration less than 48 h) or persistent (duration 48 h or more) in accordance with the Acute Disease Quality Initiative (ADQI) [39]. We did not include the KDGIO AKI criterion based on urine output, as urine output is rarely measured outside the intensive care setting [40].

BOX 1 **Applied definitions of acute kidney injury stages.**

**Table:** 

Stage	Serum creatinine criteria
1	1.5–1.9 times baseline OR ≥ 26.5 μmol/L increase in creatinine within 48 h
2	2.0–2.9 times baseline
3	≥ 3.0 times baseline OR Increase in serum creatinine to ≥ 354 μmol/L OR Initiation of acute dialysis

### 2.5 Covariates

We used CRS to obtain information on age and sex, and the patient registry to obtain information on comorbidities at any time before the start of treatment (see Table S1 for variables and codes included; see Figure 1 for visualization of the covariate assessment). Based on the identified comorbidities, we constructed a modified version of the Charlson Comorbidity Index (CCI) score, excluding cancer‐related diagnoses. We categorized the CCI score into three categories: low (score 0), moderate (score 1–2), and high (score over 2) [41]. Furthermore, we used the patient registry and the pathology registry to retrieve information on distant metastasis registered before or on the day of start of treatment based on diagnosis and pathology codes (only for solid cancers; see Table S1 for codes included).

### 2.6 Statistical Analysis

#### 2.6.1 Descriptive Analyses

For all adult cancer patients, we reported the distribution of sex, age, year of diagnosis, and baseline eGFR. We reported the distribution of sex, age, year of diagnosis, baseline eGFR, presence of distant metastases, and CCI score by cancer and treatment.

#### 2.6.2 AKI Incidence

We computed the cumulative incidence (risk) of first‐time AKI by cancer and treatment, accounting for death as a competing risk. For each surgery, we calculated the risk of AKI within the first 7 days after the first surgery in accordance with ADQI and the PeriOperative Quality Initiative definition of postoperative AKI (see Figure 1) [42]. For each treatment with anticancer drugs, we calculated the risk of AKI within the first year after the first administration based on previous studies on the risk of AKI following anticancer drug treatment [8, 9, 10]. For each HSCT, we calculated the risk of AKI within the first 100 days after the first HSCT based on previous studies' definitions of post‐HSCT AKI [43]. The treatments were analyzed regardless of the existence of other concomitant treatments; hence, patients could be included in multiple treatment groups. To examine the severity of the AKI events, we reported the proportion of stage 2–3 and persistent AKI among the AKI events.

#### 2.6.3 Mortality

We calculated the 1‐year mortality by cancer and treatment among patients with AKI following the treatment (i.e., within 7 days after surgery, 365 days after anticancer drug, and 100 days after HSCT) and comparisons without AKI following treatment matched on sex, age by decade, days since the start of treatment, cancer type, metastasis status, treatment, and CCI score (0, 1–2, +3); matched 1 to 10 with replacement (see Figure 1). The patients with AKI were followed from the day of AKI and the comparisons were followed from the corresponding day after treatment start. The comparisons were censored upon the occurrence of AKI following the treatment. We estimated the 1‐year hazard ratios (HRs) of death comparing patients with AKI with comparisons without AKI using Cox proportional hazards regression stratified by the matched groups. We generally observed higher hazards early in follow‐up; we therefore also computed the 90‐day hazard ratio [44]. The 95% confidence intervals (CIs) were computed through robust variance estimators to account for repeated comparisons.

## 3 Results

### 3.1 Patient Characteristics

Overall, we identified 357,870 adult patients with incident cancer. The median age was 70 years (first and third quartiles (Q1–Q3), 61–77), and 52.5% were male (Table 1). Among patients with a baseline outpatient eGFR available (94.1%), the median eGFR was 82 mL/min/1.73 m^2^ (Q1–Q3, 67–92). The patients' characteristics are presented by cancer and treatment in Table S2.

**TABLE 1:** Patients' characteristics at cancer diagnosis.

*N*	357,870
Age, median (Q1‐Q3)	70 (61–77)
Year, *N* (%)	
2010	14,352 (4.0)
2011	16,192 (4.5)
2012	16,885 (4.7)
2013	18,021 (5.0)
2014	22,900 (6.4)
2015	25,188 (7.1)
2016	29,906 (8.4)
2017	29,729 (8.3)
2018	29,355 (8.2)
2019	29,723 (8.3)
2020	29,430 (8.2)
2021	30,223 (8.5)
2022	30,720 (8.6)
2023	30,332 (8.5)
2024	4913 (1.4)
Male sex, *N* (%)	187,994 (52.5)
Baseline outpatient eGFR (ml/min/1.73 m^2^), median (Q1‐Q3)	82 (67–92)
Missing outpatient eGFR, *N* (%)	21,158 (5.9)

Abbreviations: eGFR, estimated glomerular filtration rate; Q1, first quartile; Q3, third quartile.

### 3.2 Acute Kidney Injury After Surgery

The 7‐day risk of AKI after surgery was lowest after breast cancer surgery (0.3%) and highest after radical nephrectomy in kidney cancer (68.9%; Table 2). Among the AKI events following surgery, the proportion of persistent AKI ranged from 15% (breast cancer surgery) to 87% (radical nephrectomy in kidney cancer), while the proportion of stage 2–3 AKI ranged from 20% (radical nephrectomy in kidney cancer) to 43% (cystectomy in urinary bladder cancer; Table S3). For the majority of surgical procedures, patients with AKI had a higher 1‐year mortality than patients without AKI (Table 2). An exception to this was radical nephrectomy in kidney cancer, which also yielded the lowest 1‐year HR (0.8 [95% CI, 0.7–1.1]). Colectomy in colon cancer (HR 3.5 [95% CI, 3.1–3.8]) and radical prostatectomy in prostate cancer (3.2 [95% CI, 1.5–6.7]) yielded the highest 1‐year HRs, yet with considerably higher 1‐year mortality after AKI following colectomy (25%) than following prostatectomy (2%).

**TABLE 2:** Risk of AKI within 7 days after surgery and 1‐year mortality in patients with and without AKI after surgery.

Cancer	Treatment	AKI		Death		
		N AKI/ N patients	7‐day risk, % (95% CI)	1‐year risk, % (95% CI)		1‐year HR, (95% CI)
				With AKI	Without AKI	
Esophagus	Esophagus res	105/1376	7.6 (6.4–9.2)	34 (24–42)	20 (16–24)	2.0 (1.4–3.0)
Stomach	Gastrectomy	128/1743	7.3 (6.2–8.7)	28 (20–36)	19 (15–22)	1.6 (1.1–2.4)
Colon	Colectomy	2419/22339	10.8 (10–11)	25 (24–27)	9 (9–10)	3.5 (3.1–3.8)
	HIPEC	27/420	6.4 (4.5–9.3)	‐^a^	‐^a^	‐^a^
Rectum	Ex of rectum	950/9253	10.3 (9.7–11)	13 (11–15)	5 (4–6)	2.6 (2.1–3.3)
Liver	Destruction of liver	47/577	8.1 (6.2–11)	29 (14–41)	22 (16–29)	1.3 (0.7–2.5)
	Liver res	80/547	14.6 (12–18)	27 (16–36)	10 (6–14)	2.9 (1.7–5.2)
Pancreas	Ex of pancreas	169/1796	9.4 (8.2–11)	35 (27–42)	27 (23–31)	1.7 (1.2–2.3)
Gall bladder	Cholecystectomy	19/209	9.1 (5.9–14)	49 (20–68)	46 (27–59)	0.9 (0.3–2.5)
Lung	Lobectomy of lung	615/7937	7.8 (7.2–8.4)	15 (12–18)	9 (7–10)	1.9 (1.5–2.5)
	Minor res of lung	134/1905	7.0 (6.0–8.3)	23 (16–30)	12 (9–15)	2.0 (1.3–3.2)
Breast	Breast surgery	103/36018	0.3 (0.2–0.3)	7 (2–12)	3 (2–4)	2.5 (1.2–5.3)
Cervix	Total hysterectomy	21/1207	1.7 (1.1–2.7)	‐^a^	‐^a^	‐^a^
Endometrium	Total hysterectomy	171/6492	2.6 (2.3–3.1)	16 (10–21)	7 (5–8)	2.5 (1.6–3.9)
	Omentectomy	68/1092	6.2 (4.9–7.8)	18 (8–27)	13 (9–17)	1.5 (0.8–2.9)
Ovary	Ex of ovary and tube	234/3362	7.0 (6.2–7.9)	17 (12–22)	11 (9–13)	1.8 (1.2–2.6)
	Omentectomy	228/3378	6.8 (6.0–7.7)	16 (11–21)	9 (7–11)	2.1 (1.4–3.0)
Kidney	Kidney res	723/1999	36.2 (34–38)	2 (1–3)	1 (0–2)	1.8 (0.8–3.7)
	Radical nephrectomy	2343/3400	68.9 (67–70)	8 (7–9)	9 (7–11)	0.8 (0.7–1.1)
Urinary bladder	Cystectomy	1108/3317	33.4 (32–35)	16 (14–18)	12 (10–14)	1.4 (1.1–1.6)
	TURB	231/5860	3.9 (3.5–4.5)	46 (39–52)	26 (23–28)	2.2 (1.7–2.7)
Prostate	Total ex of prostate	589/12056	4.9 (4.5–5.3)	2 (1–3)	0 (0–1)	3.2 (1.5–6.7)
Testis	Orchiectomy	9/1927	0.5 (0.2–0.9)	‐^a^	‐^a^	‐^a^
Brain	Brain surgery	53/4024	1.3 (1.0–1.7)	57 (42–69)	52 (46–57)	1.3 (0.8–1.9)

Abbreviations: AKI, acute kidney injury; CI, confidence interval; ex, excision; HIPEC, hyperthermic intraperitoneal chemotherapy; HR, hazard ratio; res, resection; TURB, transurethral resection of bladder. NB, patients could be included in multiple treatment groups.

^a^ Cells are masked, so it is not possible to identify or back‐calculate numbers less than 5.

### 3.3 Acute Kidney Injury After Anticancer Drug

The 1‐year risk of AKI after initiation of anticancer drugs was lowest following cyclophosphamide in breast cancer (3.5%) and highest following anticancer drugs in acute lymphatic leukemia (ALL; 79.3%; Table 3). Among the AKI events following anticancer drugs, the proportion of persistent AKI ranged from 17% (bevacizumab in brain cancer) to 59% (immune checkpoint inhibitors [ICI] in urinary bladder cancer), while the proportion of stage 2–3 AKI ranged from 8% (bleomycin + etoposide + adriamycin + cyclophosphamide + vincristine + procarbazine + prednisone in Hodgkin lymphoma) to 43% (doxorubicin in endometrium cancer; Table S4). For all anticancer drugs, patients with AKI had a higher 1‐year mortality than patients without AKI (Table 3). The 1‐year HRs were generally high, especially for treatments in non‐acute leukemic hematological malignancies, and ranged from 1.4 (95% CI, 0.7–2.7; anticancer drugs in ALL) to 40.1 (95% CI, 14–114; imatinib in chronic myeloid leukemia).

**TABLE 3:** Risk of AKI within 1 year after initiation of anticancer drug and 1‐year mortality in patients with and without AKI after anticancer drug.

Cancer	Treatment	AKI		Death		
		N AKI/ N patients	1‐year risk, % (95% CI)	1‐year risk, % (95% CI)		1‐year HR, (95% CI)
				With AKI	Without AKI	
Esophagus	Oxaliplatin	404/1481	28.1 (26–31)	67 (62–71)	45 (41–49)	3.0 (2.5–3.6)
	Docetaxel	191/666	29.2 (26–33)	62 (54–68)	33 (27–38)	3.8 (2.8–5.0)
Stomach	Oxaliplatin	555/2283	25.6 (24–28)	64 (60–68)	36 (33–39)	3.9 (3.3–4.6)
	Docetaxel	309/1153	28.4 (26–31)	56 (50–61)	25 (21–28)	5.1 (3.9–6.7)
Colon	Oxaliplatin	1092/6625	16.9 (16–18)	56 (53–59)	18 (17–19)	6.3 (5.6–7.1)
	Bevacizumab	769/3081	25.8 (24–27)	80 (77–83)	44 (42–46)	4.5 (4.0–5.1)
	Cetuximab	272/905	30.8 (28–34)	68 (62–73)	35 (31–40)	3.9 (3.0–4.9)
Rectum	Oxaliplatin	568/2624	22.3 (21–24)	46 (41–50)	16 (14–18)	4.8 (4.0–5.8)
	Bevacizumab	355/1392	26.5 (24–29)	79 (74–83)	36 (32–39)	5.4 (4.5–6.6)
	Cetuximab	125/421	30.1 (26–35)	58 (48–65)	30 (23–36)	3.0 (2.0–4.6)
Liver	Cisplatin	115/323	37.4 (32–43)	82 (73–88)	45 (34–53)	6.8 (4.2–11)
	Oxaliplatin	92/300	31.6 (27–37)	86 (77–92)	41 (30–49)	7.7 (4.9–12)
	Doxorubicin	87/278	31.7 (27–38)	59 (47–68)	36 (26–44)	2.4 (1.5–3.8)
	TKI	229/650	36.3 (33–40)	94 (90–96)	56 (50–62)	6.3 (4.8–8.1)
Pancreas	Oxaliplatin	576/2213	27.2 (25–29)	83 (80–86)	50 (47–52)	5.1 (4.4–5.9)
	Gemcitabine	1290/4532	29.3 (28–31)	88 (86–90)	56 (53–58)	4.8 (4.3–5.2)
Gall bladder	Cisplatin	161/415	41.7 (37–47)	82 (75–88)	45 (36–53)	5.4 (3.7–7.8)
	Oxaliplatin	179/528	35.0 (31–39)	88 (82–92)	49 (42–55)	5.3 (3.9–7.1)
Lung	Cisplatin	536/2916	18.6 (17–20)	49 (45–53)	19 (17–21)	3.9 (3.3–4.7)
	Carboplatin	3653/16107	23.3 (23–24)	80 (78–81)	46 (45–47)	4.1 (3.9–4.3)
	Bevacizumab	131/598	22.1 (19–26)	86 (79–91)	52 (45–58)	4.5 (3.3–6.2)
	ICI	1038/5321	20.8 (20–22)	75 (73–78)	35 (33–37)	4.7 (4.3–5.3)
	TKI	365/2186	17.1 (16–19)	78 (73–82)	42 (39–45)	3.9 (3.3–4.6)
Breast	CDK4/6i	217/1181	18.7 (17–21)	39 (32–46)	12 (9–15)	4.5 (3.2–6.3)
	Trastuzumab	276/5499	5.2 (4.6–5.8)	26 (20–31)	3 (2–4)	14.5 (9.8–22)
	Cyclophos	519/15590	3.5 (3.2–3.8)	17 (13–20)	2 (1–2)	13.1 (8.9–19)
Cervix	Cisplatin	175/1086	16.6 (14–19)	37 (29–44)	5 (3–7)	13.5 (8.3–22)
	Carboplatin	77/259	32.8 (27–40)	73 (59–82)	41 (30–50)	1.8 (1.1–2.8)
	Bevacizumab	77/230	36.0 (30–43)	80 (67–87)	29 (20–37)	4.1 (2.4–7.0)
Endometrium	Carboplatin	242/1455	17.4 (16–20)	67 (60–72)	15 (12–17)	9.4 (7.3–12)
	Doxorubicin	140/521	28.3 (25–33)	84 (76–89)	37 (31–43)	7.4 (5.2–10)
Ovary	Carboplatin	681/3722	18.8 (18–20)	49 (45–52)	16 (14–17)	5.4 (4.6–6.4)
	PARPi	84/668	13.6 (11–17)	63 (51–73)	11 (7–14)	13.8 (8.7–22)
	Bevacizumab	241/1338	19.3 (17–22)	75 (69–80)	26 (23–29)	7.6 (6.0–9.5)
	Doxorubicin	385/1623	24.9 (23–27)	85 (80–88)	33 (30–36)	7.7 (6.4–9.1)
Kidney	ICI	262/980	28.1 (25–31)	48 (41–54)	25 (21–29)	2.4 (1.8–3.2)
	TKI	321/1431	23.5 (21–26)	74 (69–79)	32 (29–36)	5.2 (4.2–6.4)
Urinary bladder	Cisplatin	794/1483	54.8 (52–57)	32 (29–35)	17 (14–20)	2.3 (1.9–2.9)
	Carboplatin	284/738	39.9 (36–44)	69 (63–74)	37 (32–42)	4.0 (3.2–5.2)
	ICI	229/719	34.9 (31–39)	77 (71–82)	31 (25–36)	6.2 (4.6–8.3)
Prostate	Docetaxel	675/4334	16.3 (15–18)	64 (60–68)	19 (17–20)	7.2 (6.3–8.3)
Testis	Cisplatin	84/791	10.9 (8.9–13)	17 (8–25)	2 (0–4)	8.6 (3.0–24)
Brain	Bevacizumab	58/1054	5.6 (4.4–7.2)	‐^a^	‐^a^	‐^a^
Melanoma	Dabra/Tram	88/367	25.7 (21–31)	69 (57–77)	36 (27–44)	4.2 (2.5–6.9)
	ICI	295/1676	18.6 (17–21)	52 (45–57)	13 (11–15)	7.1 (5.5–9.1)
	Enco/bini	48/160	30.6 (24–39)	59 (43–71)	33 (19–44)	2.6 (1.2–5.5)
Non‐Hodgkin lymphoma	CHOP/CHOEP	1071/3677	29.6 (28–31)	36 (33–39)	6 (5–7)	9.4 (7.7–12)
	Bendamustin	425/1897	23.4 (22–25)	41 (36–46)	7 (5–8)	9.9 (7.6–13)
Hodgkin lymphoma	ABVD	82/624	13.3 (11–16)	11 (4–18)	3 (0–5)	3.3 (1.1–9.6)
	BEACOPP	59/169	35.6 (29–44)	‐^a^	‐^a^	‐^a^
Multiple myeloma	Bortezomib	970/2928	34.1 (32–36)	27 (24–30)	5 (4–6)	8.7 (6.8–11)
	Daratumumab	354/1448	26.2 (24–29)	57 (51–62)	9 (7–11)	13.6 (10–18)
	Carfilzomib	227/537	44.7 (41–49)	59 (52–65)	11 (7–14)	9.3 (6.2–14)
ALL	All drugs	241/305	79.3 (75–84)	24 (18–29)	15 (5–24)	1.4 (0.7–2.7)
AML	High dose chemo	444/730	61.7 (58–65)	37 (33–42)	15 (11–19)	3.3 (2.4–4.4)
	Low dose chemo	407/807	52.0 (49–56)	66 (60–70)	37 (32–42)	3.6 (2.9–4.5)
CLL	Cyclophos	52/208	25.3 (20–32)	41 (26–53)	6 (1–10)	11.3 (3.7–34)
	Bendamustin	99/400	25.8 (22–31)	22 (13–30)	3 (0–5)	8.8 (3.0–26)
	Ibrutinib	45/293	15.5 (12–20)	54 (37–67)	4 (1–7)	25.1 (11–58)
	Venetoclax	29/312	9.8 (6.9–14)	28 (9–43)	6 (1–10)	7.4 (2.1–26)
	All drugs	290/1648	18.1 (16–20)	34 (28–39)	8 (6–10)	5.6 (4.1–7.7)
CML	Imatinib	79/601	13.5 (11–17)	33 (21–42)	1 (0–2)	40.1 (14–114)
	2nd gen TKI	47/385	12.4 (9.5–16)	33 (18–46)	4 (0–8)	10.6 (3.8–29)
	All drugs	137/764	18.4 (16–21)	42 (33–50)	5 (2–7)	16.2 (9.0–29)
Other leukemias	All drugs	448/1219	37.7 (35–41)	55 (50–60)	15 (12–17)	7.1 (5.6–9.1)

*Note:* NB: patients could be included in multiple.

Abbreviations: ABVD, adriamycin+bleomycin+vinblastine+dacarbazine; AKI, acute kidney injury; ALL, acute lymphatic leukemia; AML, acute myeloid leukemia; BEACOPP, bleomycin + etoposide + adriamycin + cyclophosphamide + vincristine + procarbazine + prednisone; CDK4/6i, Cyclin‐dependent kinase 4/6 inhibitors; chemo, chemotherapy; CHOP/CHOEP, cyclophosphamide + hydroxydaunorubicin + vincristine + (etoposide) + prednisone; CI, confidence interval; CLL, chronic lymphatic leukemia; CML, chronic myeloid leukemia; cyclophos, cyclophosphamide; dabra/tram, dabrafenib/trametinib; enco/bini, encorafenib/binimetinib; gen, generation; HR, hazard ratio; ICI, immune checkpoint inhibitors; PARPi, poly(ADP‐ribose) polymerase inhibitors; TKI, tyrosine kinase inhibitors. NB: patients could be included in multiple treatment groups.

^a^ Cells are masked, so it is not possible to identify or back‐calculate numbers less than 5.

### 3.4 Acute Kidney Injury After Hematopoietic Stem Cell Transplantation

AKI was common after HSCT, where the 100‐day risks ranged from 20.7% (autologous HSCT in multiple myeloma) to 81.8% (allogeneic HSCT in leukemia; Table 4). The proportion of persistent AKI was generally high and ranged from 55% (autologous HSCT in non‐Hodgkin lymphoma) to 76% (allogeneic HSCT in leukemia), while the proportion of stage 2–3 AKI ranged from 21% (autologous HSCT in multiple myeloma and non‐Hodgkin lymphoma) to 29% (HSCT in leukemia and Hodgkin lymphoma; Table S5). The 1‐year mortality after AKI was considerably higher in non‐Hodgkin (28%) than multiple myeloma (6%); still, patients with AKI following HSCT had a higher 1‐year mortality than patients without AKI in both non‐Hodgkin lymphoma and multiple myeloma (Table 4). The 1‐year HRs ranged from 1.1 (95% CI, 0.7–1.7; allogeneic HSCT in leukemia) to 3.8 (95% CI, 2.2–5.6; autologous HSCT in non‐Hodgkin lymphoma).

**TABLE 4:** Risk of AKI within 100 days after HSCT and 1‐year mortality in patients with and without AKI after HSCT.

Cancer	Treatment	AKI		Death		
		N AKI/ N patients	100‐day risk, % (95% CI)	1‐year risk, % (95% CI)		1‐year HR, (95% CI)
				With AKI	Without AKI	
Non‐Hodgkin lymphoma	Autologous HSCT	191/585	32.7 (29–37)	28 (21–34)	10 (6–13)	3.5 (2.2–5.6)
Hodgkin lymphoma	Autologous HSCT	22/63	35.2 (25–49)	‐^a^	‐^a^	‐^a^
Multiple myeloma	Autologous HSCT	217/1056	20.7 (18–23)	6 (3–9)	2 (1–3)	3.8 (1.8–8.2)
Leukemia	Allogeneic HSCT	533/656	81.8 (79–85)	20 (17–24)	19 (10–26)	1.1 (0.7–1.7)

Abbreviations: AKI, acute kidney injury; CI, confidence interval; HR, hazard ratio; HSCT, hematopoietic stem‐cell transplantation.

^a^ Cells are masked, so it is not possible to identify or back‐calculate numbers less than 5.

For all treatments, the 90‐day HR was higher than the 1‐year HR (Table S6).

## 4 Discussion

In patients with cancer, the risk, severity, and subsequent mortality of AKI varied substantially according to the treatment, both when examining surgery, anticancer drug treatment, and HSCT. Despite substantial variations in the mortality after AKI, patients with AKI had an increased mortality when compared with patients without AKI in most treatments. This study aimed to determine the total burden of AKI across cancer treatments in routine clinical care rather than the number of AKI events attributed to the cancer treatments. Hence, the reported risk of AKI is the sum of the treatments' effect, both positive (e.g., through reduction of tumor burden) and negative (e.g., through acute interstitial nephritis, hypovolemia, or sepsis), together with the effect of the patients' characteristics, including cancer type and stage. There was a considerable variation in the risk of AKI with several anticancer drugs across the cancer types, indicating that the underlying cancer disease and patients' characteristics had a significant impact on the risk of AKI. Furthermore, our general findings of increased mortality in cancer patients experiencing AKI could both be caused directly and indirectly, for example, through coinciding bleeding or cancer progression. We did observe that kidney cancer patients with AKI after radical nephrectomy had a similar 1‐year mortality when compared with patients without AKI. One potential explanation could be that patients without AKI after radical nephrectomy may have a larger tumor already suppressing the function of the affected kidney.

### 4.1 Interpretation

Previous studies have examined the risk of AKI after specific cancer surgeries, including nephrectomy [11], esophageal cancer surgery [12], colectomy [45], lung cancer surgery [13], and cystectomy [14, 15, 16]. Some of the studies reported risks similar to ours [12, 14]. However, the comparison with these studies is generally complicated by the variation in the definitions of AKI, for example, one study examined AKI within 2 days after the surgery [12], while another examined AKI within 90 days after surgery [15]. Some previous studies also explored the subsequent mortality [12, 45]. Murphy et al. reported that AKI following esophageal cancer surgery was not associated with an increased mortality [12], contrasting our finding of a two‐fold higher relative rate of AKI in esophageal cancer patients undergoing esophageal resection. This divergence may be explained by differences in the patients and settings, as Murphy et al. excluded patients undergoing emergency surgery; moreover, the estimates may have been affected by that Murphy et al. started the follow‐up at cancer diagnosis, thereby introducing immortal time bias, which may have underestimated the relative mortality. The risk of AKI after anticancer drug treatment by cancer type has previously been reported in a cohort study by Kitchlu et al. [30] The authors generally reported considerably lower risks of AKI than we observed. This may be explained by the authors' use of the International Classification of Diseases 10 diagnosis codes to ascertain AKI events, which is known to be less sensitive than creatinine measurements when ascertaining AKI [40, 46]. Other studies have explored the risk of AKI after specific anticancer drug treatments, including ICI [8, 17, 18], bortezomib [19], poly (ADP‐ribose) polymerase inhibitors (PARPi) [10], tyrosine kinase inhibitors (TKI) [20], cyclin‐dependent kinase (CDK) 4 and 6 inhibitors [21, 26], and dabrafenib/trametinib [9]. Some of these studies reported results similar to our results [9, 17], whereas other studies reported lower or higher risks [8, 10, 18, 19, 20, 21]. The divergent results may be explained by differences in the definition of AKI, differences in the length of follow‐up, and/or that the studies were based on data from a single center or healthcare system, which may have led to missing registration of AKI events happening outside the centers, e.g., at emergency departments at other centers. The few previous studies reporting on the mortality after AKI following anticancer drug treatment have mainly been focused on patients treated with ICI. These studies reported HRs ranging from 0.9 to 2.3 [17, 22, 23, 24, 25], which are considerably lower than the HRs of 2.4–7.1 observed in this study. One potential explanation could be differences in the definitions of AKI. Moreover, the results may have been affected by the aforementioned issues with studies from single centers/healthcare systems, which may have misclassified patients with AKI admitted to other centers as non‐AKI patients, thereby underestimating the HRs of death [17, 22, 23, 24, 25]. Furthermore, some of the studies started follow‐up at the initiation of ICI, thereby introducing immortal time bias and underestimating the HRs of death [17, 24, 25]. Previous studies have stated that a substantial proportion of AKI events observed following PARPi [10], CDK4/6 [26], and TKIs [20] were events of pseudo‐AKI, that is, GFR‐independent increase in creatinine, rather than true AKI events and that clinically significant AKI was rare [10, 26]. Though we could not separate pseudo‐AKIs from true AKIs after these treatments, our findings suggest that rapid increases in creatinine fulfilling KDIGO's definition of AKI are associated with a considerably poor prognosis, which underscores the prognostic value of rapid creatinine increase in these patients. Several studies have examined the risk of AKI after HSCT in hematological malignancies, reporting risks ranging from 10% to 92% [27, 28, 29]. The large variation may be attributed to differences in the distribution of hematological malignancies, transplantation type, definition of AKI, and the study period. A recently published single‐center study examining the mortality after AKI in patients treated with allogeneic HSCT found twice as high 1‐year mortality in patients with AKI than in our study. The difference may be explained by the difference in settings, including that the authors also included patients with other types of hematological malignancies than leukemia [27].

### 4.2 Strengths and Limitations

Our study takes advantage of the extensive Danish data from a uniform healthcare setting. The large cohort of more than 350,000 cancer patients with complete follow‐up and the comprehensive data on plasma creatinine measurements promote reliable and valid estimation of the absolute risk of AKI and mortality [47]. Furthermore, we examined the treatments with a harmonized methodological approach allowing comparison between the different treatments in each analysis. However, some limitations should be considered. First, we used procedure codes to identify treatments. Though we expect some degree of misclassification of the treatments, validation studies have shown that surgical procedures, anticancer drugs, and HSCT are generally coded with high positive predictive value in the patient registry [48, 49, 50, 51]. Second, we relied on routine clinical care data, and a potential lack of measurements of creatinine may have led to missing registration of AKI events; nevertheless, the identified AKI events represent the events the clinicians are presented with. Third, some treatment groups were rather small leading to imprecise estimates; hence, some of the estimates should be interpreted cautiously. Fourth, as stated earlier, due to the observational nature of the study, we could not establish causal associations between treatments and AKI or between AKI and mortality. Fifth, we did not include urine output in our definition of AKI, which means we may miss some AKI episodes based on low urine output. Finally, the study cohort was predominantly white and we cannot necessarily generalize the results to other ethnicities.

## 5 Conclusions

In this large cohort study, we have provided insight into the risk, severity, and prognosis of AKI following cancer treatment. The results from this study may support the identification of patients with the highest risk of AKI and the patients with the worst prognosis after AKI in clinical practice.

## Author Contributions

**Philip Vestergaard Munch:** conceptualization, data curation, funding acquisition, investigation, methodology, project administration, software, writing – original draft, writing – review and editing, formal analysis. **Mette Nørgaard:** conceptualization, investigation, methodology, writing – review and editing. **Simon Kok Jensen:** investigation, writing – review and editing. **Henrik Birn:** investigation, writing – review and editing. **Henrik Schmidt:** investigation, writing – review and editing. **Christian Fynbo Christiansen:** conceptualization, investigation, methodology, writing – review and editing.

## Ethics Statement

According to Danish law, no approval is required for non‐interventional registry‐based studies. This study was reported to the Danish Data Protection Agency through registration at Aarhus University (AU record No 2016–051‐000001/812).

## Conflicts of Interest

HB reports consultancy agreements with NOVO Nordisk, AstraZeneca, Bayer, Boehringer Ingelheim, Galapagos, GlaxoSmithKline (GSK), Alexion, Otsuka Pharmaceuticals, MSD, and Vifor Pharma as well as research funding paid to the institution from GSK and Vifor Pharma. None of these are related to our study. Department of Clinical Epidemiology, Department of Biomedicine, Department of Renal Medicine, and Department of Oncology at Aarhus University Hospital are involved in studies with funding from various companies as research grants. None of these are related to our study. No other disclosures related to this study were reported.

## Supporting information


**Data S1** Supporting Information.

**Data S2** Supporting Information.

## Data Availability

The data underlying this article were provided by the Danish Health Data Authority under license/by permission. Researchers may apply for data access through the Danish Health Data Authority.

## References

[1] D. T. Debela , S. G. Muzazu , K. D. Heraro , et al., “New Approaches and Procedures for Cancer Treatment: Current Perspectives,” Sage Open Medicine 9 (2021): 20503121211034366. 34408877 10.1177/20503121211034366 PMC8366192

[2] F. Martins , L. Sofiya , G. P. Sykiotis , et al., “Adverse Effects of Immune‐Checkpoint Inhibitors: Epidemiology, Management and Surveillance,” Nature Reviews. Clinical Oncology 16, no. 9 (2019): 563–580. 10.1038/s41571-019-0218-0 31092901

[3] F. Kroschinsky , F. Stölzel , S. von Bonin , et al., “New Drugs, New Toxicities: Severe Side Effects of Modern Targeted and Immunotherapy of Cancer and Their Management,” Critical Care (London, England) 21, no. 1 (2017): 89. 28407743 10.1186/s13054-017-1678-1 PMC5391608

[4] K. D. Jhaveri , R. Wanchoo , V. Sakhiya , D. W. Ross , and S. Fishbane , “Adverse Renal Effects of Novel Molecular Oncologic Targeted Therapies: A Narrative Review,” Kidney International Reports 2, no. 1 (2017): 108–123. 29318210 10.1016/j.ekir.2016.09.055 PMC5720524

[5] R. Wanchoo , A. Abudayyeh , M. Doshi , et al., “Renal Toxicities of Novel Agents Used for Treatment of Multiple Myeloma,” Clinical Journal of the American Society of Nephrology: CJASN 12, no. 1 (2017): 176–189. 27654928 10.2215/CJN.06100616 PMC5220662

[6] M. H. Rosner and M. A. Perazella , “Acute Kidney Injury in Patients With Cancer,” New England Journal of Medicine 376, no. 18 (2017): 1770–1781. 28467867 10.1056/NEJMra1613984

[7] C. F. Christiansen , M. B. Johansen , W. J. Langeberg , J. P. Fryzek , and H. T. Sørensen , “Incidence of Acute Kidney Injury in Cancer Patients: A Danish Population‐Based Cohort Study,” European Journal of Internal Medicine 22, no. 4 (2011): 399–406. 21767759 10.1016/j.ejim.2011.05.005

[8] H. Seethapathy , S. Zhao , D. F. Chute , et al., “The Incidence, Causes, and Risk Factors of Acute Kidney Injury in Patients Receiving Immune Checkpoint Inhibitors,” Clinical Journal of the American Society of Nephrology: CJASN 14, no. 12 (2019): 1692–1700. 31672794 10.2215/CJN.00990119 PMC6895474

[9] H. Seethapathy , M. D. Lee , I. A. Strohbehn , et al., “Clinical Features of Acute Kidney Injury in Patients Receiving Dabrafenib and Trametinib,” Nephrology, Dialysis, Transplantation: Official Publication of the European Dialysis and Transplant Association ‐ European Renal Association 37, no. 3 (2022): 507–514. 33355659 10.1093/ndt/gfaa372 PMC8875461

[10] S. Gupta , P. E. Hanna , T. Ouyang , et al., “Kidney Function in Patients With Ovarian Cancer Treated With Poly (ADP‐Ribose) Polymerase (PARP) Inhibitors,” Journal of the National Cancer Institute 115, no. 7 (2023): 831–837. 37074956 10.1093/jnci/djad070 PMC10323894

[11] A. Cho , J. E. Lee , G. Y. Kwon , et al., “Post‐Operative Acute Kidney Injury in Patients With Renal Cell Carcinoma Is a Potent Risk Factor for New‐Onset Chronic Kidney Disease After Radical Nephrectomy,” Nephrology, Dialysis, Transplantation: Official Publication of the European Dialysis and Transplant Association ‐ European Renal Association 26, no. 11 (2011): 3496–3501. 21406544 10.1093/ndt/gfr094

[12] C. F. Murphy , T. Dunne , J. A. Elliott , et al., “Acute Kidney Injury After Esophageal Cancer Surgery: Incidence, Risk Factors, and Impact on Oncologic Outcomes,” Annals of Surgery 275, no. 5 (2022): e683–e689. 32740248 10.1097/SLA.0000000000004146

[13] D. Cardinale , N. Cosentino , M. Moltrasio , et al., “Acute Kidney Injury After Lung Cancer Surgery: Incidence and Clinical Relevance, Predictors, and Role of N‐Terminal pro B‐Type Natriuretic Peptide,” Lung Cancer (New York) 123 (2018): 155–159. 10.1016/j.lungcan.2018.07.009 30089588

[14] P. T. Hanna , M. Peterson , J. Albersheim , et al., “Acute Kidney Injury Following Enhanced Recovery After Surgery in Patients Undergoing Radical Cystectomy,” Journal of Urology 204, no. 5 (2020): 982–988. 32469268 10.1097/JU.0000000000001153

[15] E. Hyllested , M. Vejlgaard , H. V. Stroomberg , S. L. Maibom , U. N. Joensen , and A. Røder , “Acute Kidney Injury Within 90 Days of Radical Cystectomy for Bladder Cancer: Incidence and Risk Factors,” Urology 182 (2023): 181–189. 37742849 10.1016/j.urology.2023.07.047

[16] T. Kwon , I. G. Jeong , C. Lee , et al., “Acute Kidney Injury After Radical Cystectomy for Bladder Cancer Is Associated With Chronic Kidney Disease and Mortality,” Annals of Surgical Oncology 23, no. 2 (2016): 686–693. 26442922 10.1245/s10434-015-4886-4

[17] A. Meraz‐Muñoz , E. Amir , P. Ng , et al., “Acute Kidney Injury Associated With Immune Checkpoint Inhibitor Therapy: Incidence, Risk Factors and Outcomes,” Journal for Immunotherapy of Cancer 8, no. 1 (2020): e000467. 32601079 10.1136/jitc-2019-000467 PMC7326260

[18] M. S. Koks , G. Ocak , B. B. M. Suelmann , et al., “Immune Checkpoint Inhibitor‐Associated Acute Kidney Injury and Mortality: An Observational Study,” PLoS One 16, no. 6 (2021): e0252978. 34101756 10.1371/journal.pone.0252978 PMC8186792

[19] S. M. Song , J. Jeon , H. R. Jang , et al., “Acute Kidney Injury in Bortezomib‐Treated Patients With Multiple Myeloma,” Nephrology, Dialysis, Transplantation: Official Publication of the European Dialysis and Transplant Association ‐ European Renal Association 38, no. 9 (2023): 2077–2085. 36662030 10.1093/ndt/gfad016

[20] M. F. Chen , G. Harada , D. Liu , et al., “Brief Report: Tyrosine Kinase Inhibitors for Lung Cancers That Inhibit MATE‐1 Can Lead to “False” Decreases in Renal Function,” Journal of Thoracic Oncology 19, no. 1 (2023): 153–159. 37748692 10.1016/j.jtho.2023.09.1444 PMC10841070

[21] B. E. Wilson , K. Mok , B. E. Kiely , R. Nguyen , and E. Moylan , “Association Between Ribociclib and Changes in Creatinine in Patients With Hormone Receptor Positive Metastatic Breast Cancer,” Internal Medicine Journal 49, no. 11 (2019): 1438–1442. 31713335 10.1111/imj.14629

[22] M. L. Baker , Y. Yamamoto , M. A. Perazella , et al., “Mortality After Acute Kidney Injury and Acute Interstitial Nephritis in Patients Prescribed Immune Checkpoint Inhibitor Therapy,” Journal for Immunotherapy of Cancer 10, no. 3 (2022): e004421. 35354588 10.1136/jitc-2021-004421 PMC8968986

[23] C. Stein , S. Burtey , J. Mancini , et al., “Acute Kidney Injury in Patients Treated With Anti‐Programmed Death Receptor‐1 for Advanced Melanoma: A Real‐Life Study in a Single‐Centre Cohort,” Nephrology, Dialysis, Transplantation: Official Publication of the European Dialysis and Transplant Association ‐ European Renal Association 36, no. 9 (2021): 1664–1674. 32941608 10.1093/ndt/gfaa137

[24] C. García‐Carro , M. Bolufer , R. Bury , et al., “Acute Kidney Injury as a Risk Factor for Mortality in Oncological Patients Receiving Checkpoint Inhibitors,” Nephrology, Dialysis, Transplantation: Official Publication of the European Dialysis and Transplant Association ‐ European Renal Association 37, no. 5 (2022): 887–894. 33547795 10.1093/ndt/gfab034

[25] Y. Shimamura , S. Watanabe , T. Maeda , K. Abe , Y. Ogawa , and H. Takizawa , “Incidence and Risk Factors of Acute Kidney Injury, and Its Effect on Mortality Among Japanese Patients Receiving Immune Check Point Inhibitors: A Single‐Center Observational Study,” Clinical and Experimental Nephrology 25, no. 5 (2021): 479–487. 33471239 10.1007/s10157-020-02008-1

[26] P. E. Hanna , I. A. Strohbehn , D. Moreno , et al., “Adverse Kidney Outcomes of CDK 4/6 Inhibitors for Metastatic Breast Cancer,” npj Breast Cancer 9, no. 1 (2023): 70. 37598278 10.1038/s41523-023-00576-5 PMC10439887

[27] K. Madsen , K. Pelletier , G. Côté , et al., “Acute Kidney Injury Within 100 Days Post Allogeneic Hematopoietic Cell Transplantation Is Associated With Increased Risk of Post‐Transplant Complications and Poor Transplant Outcomes,” Bone Marrow Transplantation 57, no. 9 (2022): 1411–1420. 35752740 10.1038/s41409-022-01744-0

[28] J. Vergara‐Cadavid , P. C. Johnson , H. T. Kim , et al., “Clinical Features of Acute Kidney Injury in the Early Post‐Transplantation Period Following Reduced‐Intensity Conditioning Allogeneic Hematopoietic Stem Cell Transplantation,” Transplantation and Cellular Therapy 29, no. 7 (2023): 455.e1–455.e9. 10.1016/j.jtct.2023.03.029 PMC10330095 37015320

[29] S. R. Kanduri , W. Cheungpasitporn , C. Thongprayoon , et al., “Incidence and Mortality of Acute Kidney Injury in Patients Undergoing Hematopoietic Stem Cell Transplantation: A Systematic Review and Meta‐Analysis,” QJM 113, no. 9 (2020): 621–632. 32101318 10.1093/qjmed/hcaa072 PMC7828586

[30] A. Kitchlu , E. McArthur , E. Amir , et al., “Acute Kidney Injury in Patients Receiving Systemic Treatment for Cancer: A Population‐Based Cohort Study,” Journal of the National Cancer Institute 111, no. 7 (2019): 727–736. 30423160 10.1093/jnci/djy167 PMC6624169

[31] M. Schmidt , S. A. J. Schmidt , K. Adelborg , et al., “The Danish Health Care System and Epidemiological Research: From Health Care Contacts to Database Records,” Clinical Epidemiology 11 (2019): 563–591. 31372058 10.2147/CLEP.S179083 PMC6634267

[32] M. Schmidt , S. A. Schmidt , J. L. Sandegaard , V. Ehrenstein , L. Pedersen , and H. T. Sørensen , “The Danish National Patient Registry: A Review of Content, Data Quality, and Research Potential,” Clinical Epidemiology 7 (2015): 449–490. 26604824 10.2147/CLEP.S91125 PMC4655913

[33] A. F. Grann , R. Erichsen , A. G. Nielsen , T. Frøslev , and R. W. Thomsen , “Existing Data Sources for Clinical Epidemiology: The Clinical Laboratory Information System (LABKA) Research Database at Aarhus University, Denmark,” Clinical Epidemiology 3 (2011): 133–138. 21487452 10.2147/CLEP.S17901 PMC3072155

[34] J. F. H. Arendt , A. T. Hansen , S. A. Ladefoged , H. T. Sørensen , L. Pedersen , and K. Adelborg , “Existing Data Sources in Clinical Epidemiology: Laboratory Information System Databases in Denmark,” Clinical Epidemiology 12 (2020): 469–475. 32547238 10.2147/CLEP.S245060 PMC7244445

[35] M. Schmidt , L. Pedersen , and H. T. Sørensen , “The Danish Civil Registration System as a Tool in Epidemiology,” European Journal of Epidemiology 29, no. 8 (2014): 541–549. 24965263 10.1007/s10654-014-9930-3

[36] R. Erichsen , T. L. Lash , S. J. Hamilton‐Dutoit , B. Bjerregaard , M. Vyberg , and L. Pedersen , “Existing Data Sources for Clinical Epidemiology: The Danish National Pathology Registry and Data Bank,” Clinical Epidemiology 2 (2010): 51–56. 20865103 10.2147/clep.s9908 PMC2943174

[37] S. Schneeweiss , J. A. Rassen , J. S. Brown , et al., “Graphical Depiction of Longitudinal Study Designs in Health Care Databases,” Annals of Internal Medicine 170, no. 6 (2019): 398–406. 30856654 10.7326/M18-3079

[38] J. A. L. N. Kellum , and P. Aspelin , “KDIGO Clinical Practice Guideline for Acute Kidney Injury,” Kidney International. Supplement 2, no. 1 (2012): 1–138.

[39] L. S. Chawla , R. Bellomo , A. Bihorac , et al., “Acute Kidney Disease and Renal Recovery: Consensus Report of the Acute Disease Quality Initiative (ADQI) 16 Workgroup,” Nature Reviews Nephrology 13, no. 4 (2017): 241–257. 28239173 10.1038/nrneph.2017.2

[40] J. J. Carrero , E. L. Fu , S. V. Vestergaard , et al., “Defining Measures of Kidney Function in Observational Studies Using Routine Health Care Data: Methodological and Reporting Considerations,” Kidney International 103, no. 1 (2023): 53–69. 36280224 10.1016/j.kint.2022.09.020

[41] M. E. Charlson , P. Pompei , K. L. Ales , and C. R. MacKenzie , “A New Method of Classifying Prognostic Comorbidity in Longitudinal Studies: Development and Validation,” Journal of Chronic Diseases 40, no. 5 (1987): 373–383. 3558716 10.1016/0021-9681(87)90171-8

[42] J. R. Prowle , L. G. Forni , M. Bell , et al., “Postoperative Acute Kidney Injury in Adult Non‐cardiac Surgery: Joint Consensus Report of the Acute Disease Quality Initiative and PeriOperative Quality Initiative,” Nature Reviews Nephrology 17, no. 9 (2021): 605–618. 33976395 10.1038/s41581-021-00418-2 PMC8367817

[43] A. Kogon and S. Hingorani , “Acute Kidney Injury in Hematopoietic Cell Transplantation,” Seminars in Nephrology 30, no. 6 (2010): 615–626. 21146126 10.1016/j.semnephrol.2010.09.009 PMC3432413

[44] M. A. Hernán , “The Hazards of Hazard Ratios,” Epidemiology 21, no. 1 (2010): 13–15. 20010207 10.1097/EDE.0b013e3181c1ea43 PMC3653612

[45] C. Slagelse , H. Gammelager , L. H. Iversen , H. T. Sørensen , and C. F. Christiansen , “Acute Kidney Injury and 1‐Year Mortality After Colorectal Cancer Surgery: A Population‐Based Cohort Study,” BMJ Open 9, no. 3 (2019): e024817. 10.1136/bmjopen-2018-024817 PMC6429863 30872545

[46] M. E. Grams , S. S. Waikar , B. MacMahon , S. Whelton , S. H. Ballew , and J. Coresh , “Performance and Limitations of Administrative Data in the Identification of AKI,” Clinical Journal of the American Society of Nephrology: CJASN 9, no. 4 (2014): 682–689. 24458075 10.2215/CJN.07650713 PMC3974361

[47] C. R. Lesko , M. P. Fox , and J. K. Edwards , “A Framework for Descriptive Epidemiology,” American Journal of Epidemiology 191, no. 12 (2022): 2063–2070. 35774001 10.1093/aje/kwac115 PMC10144679

[48] C. Vesteghem , R. F. Brøndum , U. G. Falkmer , A. Pottegård , L. Poulsen , and M. Bøgsted , “High Validity of the Danish National Patient Registry for Systemic Anticancer Treatment Registration From 2009 to 2019,” Clinical Epidemiology 13 (2021): 1085–1094. 34853537 10.2147/CLEP.S332776 PMC8628125

[49] J. L. Lund , T. Frøslev , T. Deleuran , et al., “Validity of the Danish National Registry of Patients for Chemotherapy Reporting Among Colorectal Cancer Patients Is High,” Clinical Epidemiology 5 (2013): 327–334. 24039450 10.2147/CLEP.S49773 PMC3770491

[50] T. B. Lauritsen , J. M. Nørgaard , M. E. Christensen , S. O. Dalton , and L. S. G. Østgård , “Positive Predictive Values of Hematological Procedure Codes in the Danish National Patient Registry‐A Population‐Based Validation Study,” Pharmacoepidemiology and Drug Safety 31, no. 9 (2022): 963–971. 35638368 10.1002/pds.5485 PMC9545071

[51] M. O. Broe , P. B. Jensen , T. O. Mattsson , and A. Pottegård , “Validity of Antineoplastic Procedure Codes in the Danish National Patient Registry: The Case of Colorectal Cancer,” Epidemiology 31, no. 4 (2020): 599–603. 32483069 10.1097/EDE.0000000000001208

