# Supplementary material for: Risk of and Mortality After Acute Kidney Injury Following Cancer Treatment: A Cohort Study
Source: Cancer Med. 2025 Feb 10;14(3):e70646. doi: 10.1002/cam4.70646 (PMC11808929; doi:10.1002/cam4.70646)
Supplement: Supplementary file 1 — Data S1 Supporting Information. [file CAM4-14-e70646-s001.docx]

**SUPPLEMENTARY MATERIAL**

**Content:
Supplementary Table 1.** Coding appendix.
**Supplementary Table 2**. Patients’ characteristics by cancer and treatment.
**Supplementary Table 3.** Distribution of stage and duration of AKI events after surgery.
**Supplementary Table 4.** Distribution of stage and duration of AKI events after anticancer drugs.
**Supplementary Table 5.** Distribution of stage and duration of AKI events after HSCT.
**Supplementary Table 6.** Ninety-day HR of death by cancer and treatment.

**Supplementary Table 1.** Coding appendix.

| **Condition** | **Source/coding system** | **Inclusion codes** |
| --- | --- | --- |
| **Cancer diagnosis** |  |  |
| Prostate | The patient registry/ ICD-10 | C61 |
| Urinary bladder | The patient registry/ ICD-10 | C67 |
| Kidney | The patient registry/ ICD-10 | C64 |
| Ovary | The patient registry/ ICD-10 | C56, C570, C571, C572, C573, C574 |
| Endometrium | The patient registry/ ICD-10 | C54, C55 |
| Cervix | The patient registry/ ICD-10 | C53 |
| Testis | The patient registry/ ICD-10 | C62 |
| Colon | The patient registry/ ICD-10 | C18, C19 |
| Rectum | The patient registry/ ICD-10 | C20 |
| Stomach | The patient registry/ ICD-10 | C16 |
| Esophagus | The patient registry/ ICD-10 | C15 |
| Liver | The patient registry/ ICD-10 | C22 |
| Gall bladder | The patient registry/ ICD-10 | C23, C24 |
| Pancreas | The patient registry/ ICD-10 | C25 |
| Breast | The patient registry/ ICD-10 | C50 |
| Lung | The patient registry/ ICD-10 | C33, C34 |
| Melanoma | The patient registry/ ICD-10 | C43 |
| Hodgkin lymphoma | The patient registry/ ICD-10 | C81 |
| Non-Hodgkin lymphoma | The patient registry/ ICD-10 | C82, C83, C84, C85, C86, C88 |
| ALL | The patient registry/ ICD-10 | C910 |
| CLL | The patient registry/ ICD-10 | C911 |
| AML | The patient registry/ ICD-10 | C920, C923, C924, C925, C926, C928 |
| CML | The patient registry/ ICD-10 | C921, C922 |
| Leukemia | The patient registry/ ICD-10 | C910, C911, C920, C921, C913, C914, C915, C916, C917, C918, C919, C922, C923, C924, C925, C926, C927, C928, C929, C93, C94, C95 |
| Other leukemias | The patient registry/ ICD-10 | C913, C914, C915 C916, DC917, C918, C919, C922, C927, C929, C93, C94, C95 |
| Multiple myeloma | The patient registry/ ICD-10 | C900 |
| Brain | The patient registry/ ICD-10 | C71 |
| **Surgical procedures** |  |  |
| Cholecystectomy | The patient registry/NCSP | KJKA2 |
| Colectomy | The patient registry/NCSP | KJFB, KJFH |
| Cystectomy | The patient registry/NCSP | KKCC |
| Destruction of liver | The patient registry/NCSP | KJJA43 |
| Res of esophagus | The patient registry/NCSP | KJCC |
| Salpingo-oophorectomy | The patient registry/NCSP | KLAF |
| Ex of pancreas | The patient registry/NCSP | KJLC |
| Ex of rectum | The patient registry/NCSP | KJGB |
| Gastrectomy | The patient registry/NCSP | KJDC, KJDD, KJDE |
| Partial res of kidney | The patient registry/NCSP | KKAD |
| Lobectomy of lung | The patient registry/NCSP | KGDC |
| Minor res of lung | The patient registry/NCSP | KGDB |
| Radical nephrectomy | The patient registry/NCSP | KKAC |
| Orchiectomy | The patient registry/NCSP | KKFC0, KKFC1 |
| Breast surgery | The patient registry/NCSP | KHAC25, KHAB40, KHAC20 |
| Brain surgery | The patient registry/NCSP | KAAG, KAAB |
| Radical prostatectomy | The patient registry/NCSP | KKEC |
| Total hysterectomy | The patient registry/NCSP | KLCD |
| TURB | The patient registry/NCSP | KKCD32 |
| HIPEC | The patient registry/NCSP | KJAQ1 |
| Omentectomy | The patient registry/NCSP | KJAL3 |
| Res of liver | The patient registry/NCSP | KJJB |
| **Anticancer drugs** |  |  |
| Cisplatin | The patient registry/Procedure | BWHA107, BWHA126, BWHA128, BWHA132, BWHA133, BWHA140, BWHA184, BWHA185, BWHA201, BWHA206, BWHA207, BWHA209, BWHA214, BWHA224, BWHA225, BWHA226, BWHA240, BWHA251, BWHA261 |
| Carboplatin | The patient registry/Procedure | BWHA109, BWHA112, BWHA127, BWHA129, BWHA130, BWHA157, BWHA203, BWHA214, BWHA224, BWHA238, BWHA242, BWHA252 |
| Oxaliplatin | The patient registry/Procedure | BWHA108, BWHA222, BWHA223, BWHA231, BWHA234, BWHA253, BWHA254 |
| Gemcitabine | The patient registry/Procedure | BWHA114, BWHA128, BWHA129, BWHA170, BWHA206, BWHA211, BWHA235, BWHA236, BWHA238, BWHA253, BWHA259 |
| PARPi | The patient registry/Procedure | BWHA433, BWHA437 |
| CDK4/6i | The patient registry/Procedure | BWHA442, BWHA444 |
| Bevacizumab | The patient registry/Procedure | BOHJ19B1 |
| Trastuzumab | The patient registry/Procedure | BOHJ13 |
| Cyclophos | The patient registry/Procedure | BWHA105, BWHA117, BWHA118, BWHA119, BWHA134, BWHA139, BWHA144, BWHA156, BWHA160, BWHA164, BWHA165, BWHA166, BWHA174, BWHA175, BWHA176, BWHA218, BWHA241, BWHA247, BWHA311, BWHA312 |
| CHOP/CHOEP | The patient registry/Procedure | BWHA165, BWHA119 |
| Bendamustin | The patient registry/Procedure | BWHA177 |
| ABVD | The patient registry/Procedure | BWHA167 |
| Dabra/tram | The patient registry/Procedure | BWHA419, BWHA420 |
| Bortezomib | The patient registry/Procedure | BWHA402 |
| Daratumumab | The patient registry/Procedure | BOHJ19H8 |
| Carfilzomib | The patient registry/Procedure | BWHA432 |
| Docetaxel | The patient registry/Procedure | BWHA208, BWHA209, BWHA210, BWHA211, BWHA247, BWHA252 |
| Doxorubicin | The patient registry/Procedure | BWHA102, BWHA119, BWHA144, BWHA146, BWHA165, BWHA170, BWHA183, BWHA184, BWHA185, BWHA216, BWHA232, BWHA237, BWHA258, BWHA259, BWHA260 |
| Cetuximab | The patient registry/Procedure | BOHJ17 |
| ICI | The patient registry/Procedure | BOHJ19H2, BOHJ19H7, BOHJ19J, BOHJ19D |
| TKI | The patient registry/Procedure | BWHA401, BWHA404, BWHA407, BWHA406, BWHA410, BWHA413, BWHA426, BWHA424, BWHA422, BWHA420, BWHA417, BWHA405, BWHA440, BWHA434, BWHA414, BWHP120, BWHA447, BWHA448, BWHA44, BWHA46, BWHA47, BWHA427 |
| Enco/bini | The patient registry/Procedure | BWHA446, BWHA447 |
| All drugs | The patient registry/Procedure | BWHA, BWHB1, BWHB2, BWHB3, BWHB8, BOHJ1 |
| High dose chemo | The patient registry/Procedure | BWHA30 |
| Low dose chemo | The patient registry/Procedure | BWHA158, BWHA256 |
| Imatinib | The patient registry/Procedure | BWHA401 |
| 2nd gen TKI | The patient registry/Procedure | BWHA411, BWHA409, BWHA425 |
| Ibrutinib | The patient registry/Procedure | BWHA427 |
| Venetoclax | The patient registry/Procedure | BWHA438 |
| BEACOPP | The patient registry/Procedure | BWHA138 |
| **HSCT** |  |  |
| Autologous HSCT | The patient registry/Procedure | BOQE1, BOQE2, BOQF0 |
| Allogeneic HSCT | The patient registry/Procedure | BOQE3, BOQE4, BOQE5, BOQE6, BOQF1, BOQF2 |
| **Dialysis** |  |  |
| Chronic dialysis | The patient registry/Procedure | BJFD2 |
| Acute dialysis | The patient registry/Procedure | BJFD0 |
| **Charlson comorbidity index score** |  |  |
| Myocardial infarction | The patient registry/ICD-10 | I21, I22, I23 |
| Congestive heart failure | The patient registry/ICD-10 | I50, I110, I130, I132 |
| Peripheral vascular disease | The patient registry/ICD-10 | I70, I71, I72, I73, I74, I77 |
| Cerebrovascular disease | The patient registry/ICD-10 | I60-I69, G45, G46 |
| Dementia | The patient registry/ICD-10 | F00-F03, F05.1, G30 |
| Chronic pulmonary disease | The patient registry/ICD-10 | J40-J47, J60-J67, J68.4, J701,  J703, J841, J920, J961, J982,  J983 |
| Connective tissue disorder | The patient registry/ICD-10 | M05, M06, M08, M09, M30, M31, M32, M33, M34, M35, M36, D86 |
| Ulcer disease | The patient registry/ICD-10 | K22.1, K25-K28 |
| Mild liver disease | The patient registry/ICD-10 | B18, K700-K703, K709, K71,  K73, K74, K760 |
| Diabetes mellitus | The patient registry/ICD-10 | E100, E101, E109, E110, E111, E119 |
| Hemiplegia | The patient registry/ICD-10 | G81, G82 |
| Moderate to severe renal  disease | The patient registry/ICD-10 | I12, I13, N00-N05, N07, N11, N14, N17-N19, Q61 |
| Diabetes with end organ damage | The patient registry/ICD-10 | E102-E108, E112-E118 |
| Moderate to severe liver disease | The patient registry/ICD-10 | B150, B160, B162, B190, K704, K72, K766, I85 |
| AIDS | The patient registry/ICD-10 | B21-B24 |
| **Metastasis** |  |  |
| Distant metastasis | The patient registry/ICD-10 | C78, C79, CxxxM |
|  | The pathology registry/SNOMED | M8xxx6, M9xxx6 |

Abbreviations: ABVD, adriamycin+bleomycin+vinblastine+dacarbazine; AIDS, acquired immunodeficiency syndrome; ALL, acute lymphatic leukemia; AML, acute myeloid leukemia; BEACOPP, bleomycin+etoposide+adriamycin+cyclophosphamide+vincristine+procarbazine+prednisone; CDK4/6i, Cyclin-dependent kinase 4/6 inhibitors; chemo, chemotherapy; CHOP/CHOEP, cyclophosphamide+hydroxydaunorubicin+vincristine+(etoposide)+prednisone ; CLL, chronic lymphatic leukemia; CML, chronic myeloid leukemia; cyclophos, cyclophosphamide; dabra/tram, dabrafenib/trametinib; enco/bini, encorafenib/binimetinib; ex, excision; gen, generation; HIPEC, hyperthermic intraperitoneal chemotherapy; HSCT, hematopoietic stem-cell transplantation; ICD, International Classification of Diseases ; ICI, immune checkpoint inhibitors; NCSP, Nordic Medico-Statistical Committee Classification of Surgical Procedures; PARPi, poly(ADP-ribose) polymerase inhibitors; res, resection; SNOMED, Systematized Nomenclature of Medicine; TKI, tyrosine kinase inhibitors. TURB, transurethral resection of bladder.
NB: patients could be included in multiple treatment groups.

**Supplementary Table 2.** Patients’ characteristics by cancer and treatment.

| **Cancer** | **Treatment** | **N patients** | **Age, median (Q1-Q3)** | **Female sex, %** | **eGFR (ml/min/1.73 m^2^), median (Q1-Q3)** | **Low/moderate/high CCIs, %** | **Distant metastasis, %** |
| --- | --- | --- | --- | --- | --- | --- | --- |
| Esophagus | Esophagus res | 1376 | 67 (61-73) | 22.0 | 86 (72-94) | 58/35/7 | 48 |
| Esophagus | Oxaliplatin | 1481 | 67 (61-73) | 20.0 | 86 (74-94) | 62/32/6 | 42 |
| Esophagus | Docetaxel | 666 | 66 (60-72) | 20.0 | 86 (74-94) | 61/34/5 | 29 |
| Stomach | Gastrectomy | 1743 | 68 (60-75) | 36.0 | 83 (70-92) | 50/41/8 | 44 |
| Stomach | Oxaliplatin | 2283 | 67 (59-73) | 27.0 | 85 (72-94) | 60/35/5 | 43 |
| Stomach | Docetaxel | 1153 | 65 (57-71) | 26.0 | 86 (74-94) | 62/34/5 | 31 |
| Colon | Colectomy | 22339 | 72 (64-79) | 50.0 | 80 (66-89) | 59/33/8 | 12 |
| Colon | HIPEC | 420 | 62 (53-69) | 60.0 | 89 (81-97) | 74/22/4 | 93 |
| Colon | Oxaliplatin | 6625 | 65 (58-71) | 46.0 | 86 (75-94) | 72/24/3 | 40 |
| Colon | Bevacizumab | 3081 | 68 (60-75) | 46.0 | 84 (71-92) | 70/25/4 | 81 |
| Colon | Cetuximab | 905 | 66 (59-72) | 43.0 | 86 (74-94) | 70/26/5 | 78 |
| Rectum | Ex of rectum | 9253 | 69 (61-75) | 37.0 | 84 (71-93) | 68/28/5 | 10 |
| Rectum | Oxaliplatin | 2624 | 64 (55-69) | 36.0 | 89 (78-97) | 76/21/3 | 44 |
| Rectum | Bevacizumab | 1392 | 66 (59-73) | 33.0 | 87 (75-95) | 69/27/4 | 77 |
| Rectum | Cetuximab | 421 | 63 (56-69) | 33.0 | 89 (78-97) | 74/22/4 | 70 |
| Liver | Destruction of liver | 577 | 67 (60-73) | 22.0 | 88 (72-97) | 25/29/46 | 7 |
| Liver | Liver res | 547 | 68 (60-74) | 33.0 | 88 (75-96) | 44/33/23 | 15 |
| Liver | Cisplatin | 323 | 66 (58-73) | 48.0 | 90 (77-97) | 58/33/9 | 52 |
| Liver | Oxaliplatin | 300 | 66 (58-72) | 50.0 | 87 (74-96) | 60/31/9 | 59 |
| Liver | Doxorubicin | 278 | 68 (61-74) | 19.0 | 89 (75-99) | 31/24/45 | 9 |
| Liver | TKI | 650 | 68 (62-74) | 18.0 | 87 (73-96) | 39/26/35 | 29 |
| Pancreas | Ex of pancreas | 1796 | 68 (60-74) | 45.0 | 89 (77-97) | 62/32/6 | 41 |
| Pancreas | Oxaliplatin | 2213 | 65 (58-70) | 44.0 | 92 (83-99) | 69/26/5 | 59 |
| Pancreas | Gemcitabine | 4532 | 69 (63-75) | 47.0 | 88 (77-96) | 62/31/7 | 58 |
| Gall bladder | Cholecystectomy | 209 | 69 (62-75) | 57.0 | 83 (67-93) | 54/33/12 | 33 |
| Gall bladder | Cisplatin | 415 | 66 (58-72) | 62.0 | 90 (77-98) | 70/24/6 | 60 |
| Gall bladder | Oxaliplatin | 528 | 67 (60-73) | 53.0 | 88 (75-96) | 67/26/8 | 62 |
| Lung | Lobectomy of lung | 7937 | 69 (63-75) | 54.0 | 85 (71-93) | 49/41/9 | 23 |
| Lung | Minor res of lung | 1905 | 70 (64-76) | 59.0 | 83 (67-92) | 37/49/13 | 21 |
| Lung | Cisplatin | 2916 | 64 (57-69) | 54.0 | 91 (81-98) | 63/33/4 | 64 |
| Lung | Carboplatin | 16107 | 69 (62-74) | 49.0 | 87 (75-95) | 51/41/8 | 76 |
| Lung | Bevacizumab | 598 | 65 (59-71) | 58.0 | 90 (79-98) | 63/34/4 | 85 |
| Lung | ICI | 5321 | 68 (62-74) | 51.0 | 88 (76-95) | 54/39/7 | 85 |
| Lung | TKI | 2186 | 68 (61-75) | 60.0 | 86 (74-95) | 62/32/6 | 84 |
| Breast | Breast surgery | 36018 | 64 (54-73) | 99.0 | 86 (74-95) | 72/25/3 | 37 |
| Breast | CDK4/6i | 1181 | 65 (53-74) | 99.0 | 84 (71-95) | 72/23/5 | 87 |
| Breast | Trastuzumab | 5499 | 59 (49-68) | 99.0 | 90 (78-99) | 77/21/2 | 39 |
| Breast | Cyclophos | 15590 | 54 (47-63) | 100.0 | 92 (81-101) | 80/18/2 | 41 |
| Cervix | Total hysterectomy | 1207 | 46 (39-59) | 100.0 | 95 (83-107) | 77/21/2 | 11 |
| Cervix | Cisplatin | 1086 | 51 (41-62) | 100.0 | 94 (82-105) | 78/20/2 | 19 |
| Cervix | Carboplatin | 259 | 58 (45-68) | 100.0 | 85 (69-97) | 67/30/3 | 54 |
| Cervix | Bevacizumab | 230 | 52 (43-63) | 100.0 | 89 (76-102) | 72/25/3 | 63 |
| Endometrium | Total hysterectomy | 6492 | 69 (61-76) | 100.0 | 80 (66-91) | 69/27/5 | 14 |
| Endometrium | Omentektomy | 1092 | 70 (63-75) | 100.0 | 82 (69-91) | 71/26/3 | 42 |
| Endometrium | Carboplatin | 1455 | 69 (62-75) | 100.0 | 82 (68-91) | 70/27/3 | 70 |
| Endometrium | Doxorubicin | 521 | 67 (60-72) | 100.0 | 84 (70-93) | 68/28/4 | 79 |
| Ovary | Ex of ovary and tube | 3362 | 65 (56-73) | -^a^ | 85 (72-95) | 73/24/3 | 70 |
| Ovary | Omentektomy | 3378 | 65 (56-73) | -^a^ | 86 (73-95) | 74/24/2 | 70 |
| Ovary | Carboplatin | 3722 | 68 (58-75) | 100.0 | 84 (70-93) | 72/25/3 | 68 |
| Ovary | PARPi | 668 | 63 (55-71) | 100.0 | 87 (75-96) | 73/25/2 | 93 |
| Ovary | Bevacizumab | 1338 | 66 (58-73) | 100.0 | 85 (73-93) | 73/25/2 | 89 |
| Ovary | Doxorubicin | 1623 | 66 (58-72) | 100.0 | 84 (71-93) | 71/26/3 | 89 |
| Kidney | Kidney res | 1999 | 63 (55-70) | 32.0 | 87 (71-96) | 59/33/8 | 2 |
| Kidney | Radical nephrectomy | 3400 | 66 (58-73) | 33.0 | 81 (66-93) | 62/30/8 | 14 |
| Kidney | ICI | 980 | 64 (57-70) | 26.0 | 82 (67-94) | 65/29/6 | 87 |
| Kidney | TKI | 1431 | 65 (58-72) | 27.0 | 80 (64-92) | 61/30/9 | 83 |
| Urinary bladder | Cystectomy | 3317 | 70 (63-75) | 26.0 | 79 (64-90) | 63/32/5 | 20 |
| Urinary bladder | TURB | 5860 | 74 (67-80) | 24.0 | 75 (58-87) | 52/37/11 | 3 |
| Urinary bladder | Cisplatin | 1483 | 66 (60-71) | 24.0 | 85 (70-93) | 70/27/3 | 28 |
| Urinary bladder | Carboplatin | 738 | 72 (66-76) | 26.0 | 71 (56-87) | 54/38/8 | 59 |
| Urinary bladder | ICI | 719 | 70 (63-75) | 24.0 | 78 (61-90) | 57/35/8 | 73 |
| Prostate | Total ex of prostate | 12056 | 66 (61-70) | 0.0 | 84 (74-91) | 76/22/2 | 9 |
| Prostate | Docetaxel | 4334 | 70 (65-75) | 0.0 | 82 (70-90) | 66/29/5 | 72 |
| Testis | Orchiectomy | 1927 | 37 (29-47) | 0.0 | 103 (91-112) | 87/12/1 | 5 |
| Testis | Cisplatin | 791 | 35 (28-44) | -^a^ | 105 (93-114) | 87/-^a^/-^a^ | 51 |
| Brain | Brain surgery | 4024 | 63 (54-71) | 40.0 | 85 (73-95) | 68/28/4 | 6 |
| Brain | Bevacizumab | 1054 | 57 (48-66) | 38.0 | 89 (78-98) | 73/25/2 | 4 |
| Melanoma | Dabra/Tram | 367 | 65 (53-74) | 39.0 | 85 (74-95) | 58/35/7 | 96 |
| Melanoma | ICI | 1676 | 67 (57-75) | 39.0 | 84 (71-93) | 66/30/5 | 95 |
| Melanoma | Enco/bini | 160 | 63 (54-71) | 39.0 | 86 (72-94) | 57/38/5 | 94 |
| Non-Hodgkin lymphoma | CHOP/CHOEP | 3677 | 68 (59-76) | 42.0 | 83 (68-93) | 63/32/6 | NA |
| Non-Hodgkin lymphoma | Bendamustin | 1897 | 69 (61-75) | 43.0 | 81 (67-91) | 60/34/7 | NA |
| Non-Hodgkin lymphoma | Autologous HSCT | 585 | 58 (51-64) | 32.0 | 91 (80-100) | 73/23/4 | NA |
| Hodgkin lymphoma | ABVD | 624 | 45 (27-63) | 41.0 | 100 (87-115) | 72/24/4 | NA |
| Hodgkin lymphoma | Autologous HSCT | 63 | 43 (27-59) | 29.0 | 104 (90-118) | 70/-^a^/-^a^ | NA |
| Hodgkin lymphoma | BEACOPP | 169 | 28 (22-37) | 40.0 | 116 (107-126) | 85/-^a^/-^a^ | NA |
| Multiple myeloma | Bortezomib | 2928 | 70 (62-76) | 43.0 | 75 (58-88) | 61/31/8 | NA |
| Multiple myeloma | Daratumumab | 1448 | 71 (63-76) | 43.0 | 75 (59-88) | 53/36/11 | NA |
| Multiple myeloma | Carfilzomib | 537 | 64 (57-71) | 42.0 | 78 (64-93) | 62/30/8 | NA |
| Multiple myeloma | Autologous HSCT | 1056 | 61 (55-65) | 42.0 | 83 (66-95) | 71/26/3 | NA |
| Leukemia | Allogeneic HSCT | 656 | 56 (43-64) | 42.0 | 90 (77-99) | 73/25/2 | NA |
| ALL | All drug | 305 | 46 (28-65) | 39.0 | 86 (68-100) | 74/22/4 | NA |
| AML | High dose chemo | 730 | 60 (49-68) | 44.0 | 88 (74-98) | 75/22/3 | NA |
| AML | Low dose chemo | 807 | 72 (63-78) | 39.0 | 79 (65-89) | 55/36/8 | NA |
| CLL | Cyclophos | 208 | 63 (56-69) | 30.0 | 82 (72-92) | 68/27/5 | NA |
| CLL | Bendamustin | 400 | 68 (63-74) | 28.0 | 78 (65-88) | 66/28/6 | NA |
| CLL | Ibrutinib | 293 | 68 (60-75) | 29.0 | 77 (64-88) | 63/28/9 | NA |
| CLL | Venetoclax | 312 | 66 (58-73) | 27.0 | 81 (68-91) | 58/36/6 | NA |
| CLL | All drugs | 1648 | 69 (62-76) | 34.0 | 78 (64-89) | 58/35/7 | NA |
| CML | Imatinib | 601 | 59 (46-70) | 45.0 | 84 (72-97) | 68/25/7 | NA |
| CML | 2nd gen TKI | 385 | 56 (44-67) | 44.0 | 86 (72-99) | 67/26/7 | NA |
| CML | All drug | 764 | 61 (48-72) | 44.0 | 84 (70-97) | 68/24/7 | NA |
| Other leukemias | All drugs | 1219 | 70 (61-78) | 37.0 | 78 (61-89) | 54/37/9 | NA |

^a^Cells are masked, so it is not possible to identify or back-calculate numbers less than 5.
Abbreviations: ABVD, adriamycin+bleomycin+vinblastine+dacarbazine; ALL, acute lymphatic leukemia; AML, acute myeloid leukemia; BEACOPP, bleomycin+etoposide+adriamycin+cyclophosphamide+vincristine+procarbazine+prednisone; CCIs, Charlson Comorbidity Index score; CDK4/6i, Cyclin-dependent kinase 4/6 inhibitors; chemo, chemotherapy; CHOP/CHOEP, cyclophosphamide+hydroxydaunorubicin+vincristine+(etoposide)+prednisone ; CLL, chronic lymphatic leukemia; CML, chronic myeloid leukemia; cyclophos, cyclophosphamide; dabra/tram, dabrafenib/trametinib; eGFR, estimated glomerular filtration rate; enco/bini, encorafenib/binimetinib; ex, excision; gen, generation; HIPEC, hyperthermic intraperitoneal chemotherapy; HSCT, hematopoietic stem-cell transplantation; ICI, immune checkpoint inhibitors; PARPi, poly(ADP-ribose) polymerase inhibitors; Q1, first quartile; Q3 third quartile; res, resection; TKI, tyrosine kinase inhibitors. TURB, transurethral resection of bladder.
NB: patients could be included in multiple treatment groups.

**Supplementary Table 3.** Distribution of stage and duration of AKI events after surgery.

| **Cancer** | **Treatment** | **Persistent AKI, %** | **Stage 2-3 AKI, %** |
| --- | --- | --- | --- |
| Esophagus | Esophagus res | 35 | 30 |
| Stomach | Gastrectomy | 39 | 30 |
| Colon | Colectomy | 35 | 36 |
| Colon | HIPEC | -^a^ | 33 |
| Rectum | Ex of rectum | 39 | 37 |
| Liver | Destruction of liver | 43 | 26 |
| Liver | Liver res | 48 | 31 |
| Pancreas | Ex of pancreas | 33 | 31 |
| Gall bladder | Cholecystectomy | -^a^ | 37 |
| Lung | Lobectomy of lung | 37 | 28 |
| Lung | Minor res of lung | 44 | 29 |
| Breast | Breast surgery | 15 | 28 |
| Cervix | Total hysterectomy | 24 | 24 |
| Endometrium | Total hysterectomy | 22 | 31 |
| Endometrium | Omentectomy | 18 | 34 |
| Ovary | Ex of ovary and tube | 29 | 28 |
| Ovary | Omentectomy | 28 | 27 |
| Kidney | Kidney res | 55 | 22 |
| Kidney | Radical nephrectomy | 87 | 20 |
| Urinary bladder | Cystectomy | 51 | 43 |
| Urinary bladder | TURB | 51 | 30 |
| Prostate | Total ex of prostate | 22 | 27 |
| Testis | Orchiectomy | -^a^ | -^a^ |
| Brain | Brain surgery | 36 | 21 |

^a^Cells are masked, so it is not possible to identify or back-calculate numbers less than 5.
Abbreviations: AKI, acute kidney injury; ex, excision; HIPEC, hyperthermic intraperitoneal chemotherapy; res, resection; TURB, transurethral resection of bladder.
NB: patients could be included in multiple treatment groups.

**Supplementary Table 4.** Distribution of stage and duration of AKI events after anticancer drugs.

| **Cancer** | **Treatment** | **Persistent AKI, %** | **Stage 2-3 AKI, %** |
| --- | --- | --- | --- |
| Esophagus | Oxaliplatin | 35 | 30 |
| Esophagus | Docetaxel | 32 | 25 |
| Stomach | Oxaliplatin | 40 | 31 |
| Stomach | Docetaxel | 37 | 30 |
| Colon | Oxaliplatin | 38 | 33 |
| Colon | Bevacizumab | 37 | 34 |
| Colon | Cetuximab | 32 | 33 |
| Rectum | Oxaliplatin | 40 | 35 |
| Rectum | Bevacizumab | 39 | 36 |
| Rectum | Cetuximab | 46 | 41 |
| Liver | Cisplatin | 33 | 31 |
| Liver | Oxaliplatin | 43 | 38 |
| Liver | Doxorubicin | 44 | 34 |
| Liver | TKI | 43 | 39 |
| Pancreas | Oxaliplatin | 32 | 33 |
| Pancreas | Gemcitabine | 34 | 34 |
| Gall bladder | Cisplatin | 37 | 33 |
| Gall bladder | Oxaliplatin | 36 | 42 |
| Lung | Cisplatin | 43 | 26 |
| Lung | Carboplatin | 33 | 28 |
| Lung | Bevacizumab | 31 | 27 |
| Lung | ICI | 37 | 26 |
| Lung | TKI | 33 | 28 |
| Breast | CDK4/6i | 57 | 23 |
| Breast | Trastuzumab | 40 | 30 |
| Breast | Cyclophos | 33 | 24 |
| Cervix | Cisplatin | 49 | 29 |
| Cervix | Carboplatin | 52 | 42 |
| Cervix | Bevacizumab | 49 | 32 |
| Endometrium | Carboplatin | 42 | 31 |
| Endometrium | Doxorubicin | 45 | 43 |
| Ovary | Carboplatin | 42 | 26 |
| Ovary | PARPi | 39 | 30 |
| Ovary | Bevacizumab | 41 | 23 |
| Ovary | Doxorubicin | 44 | 29 |
| Kidney | ICI | 58 | 39 |
| Kidney | TKI | 46 | 33 |
| Urinary bladder | Cisplatin | 57 | 35 |
| Urinary bladder | Carboplatin | 51 | 36 |
| Urinary bladder | ICI | 59 | 36 |
| Prostate | Docetaxel | 50 | 32 |
| Testis | Cisplatin | 48 | 30 |
| Brain | Bevacizumab | 17 | 21 |
| Melanoma | Dabra/Tram | 39 | 27 |
| Melanoma | ICI | 53 | 37 |
| Melanoma | Enco/bini | 35 | 29 |
| Non-Hodgkin lymphoma | CHOP/CHOEP | 49 | 23 |
| Non-Hodgkin lymphoma | Bendamustin | 42 | 24 |
| Hodgkin lymphoma | ABVD | 41 | 24 |
| Hodgkin lymphoma | BEACOPP | 31 | 8 |
| Multiple myeloma | Bortezomib | 50 | 31 |
| Multiple myeloma | Daratumumab | 45 | 28 |
| Multiple myeloma | Carfilzomib | 51 | 32 |
| ALL | All drug | 45 | 25 |
| AML | High dose chemo | 56 | 25 |
| AML | Low dose chemo | 49 | 21 |
| CLL | Cyclophos | 46 | 21 |
| CLL | Bendamustin | 51 | 30 |
| CLL | Ibrutinib | 40 | 29 |
| CLL | Venetoclax | 38 | 17 |
| CLL | All drugs | 45 | 32 |
| CML | Imatinib | 52 | 24 |
| CML | 2nd gen TKI | 55 | 19 |
| CML | All drugs | 52 | 23 |
| Other leukemias | All drugs | 52 | 29 |

Abbreviations: ABVD, adriamycin+bleomycin+vinblastine+dacarbazine; AKI, acute kidney injury; BEACOPP, bleomycin+etoposide+adriamycin+cyclophosphamide+vincristine+procarbazine+prednisone; ALL, acute lymphatic leukemia; AML, acute myeloid leukemia; CDK4/6i, Cyclin-dependent kinase 4/6 inhibitors; chemo, chemotherapy; CHOP/CHOEP, cyclophosphamide+hydroxydaunorubicin+vincristine+(etoposide)+prednisone ; CLL, chronic lymphatic leukemia; CML, chronic myeloid leukemia; cyclophos, cyclophosphamide; dabra/tram, dabrafenib/trametinib; enco/bini, encorafenib/binimetinib; gen, generation; ICI, immune checkpoint inhibitors; PARPi, poly(ADP-ribose) polymerase inhibitors; TKI, tyrosine kinase inhibitors.
NB: patients could be included in multiple treatment groups.

**Supplementary Table 5.** Distribution of stage and duration of AKI events after HSCT.

| **Cancer** | **Treatment** | **Persistent AKI, %** | **Stage 2-3 AKI, %** |
| --- | --- | --- | --- |
| Non-Hodgkin lymphoma | Autologous HSCT | 54 | 21 |
| Hodgkin lymphoma | Autologous HSCT | 64 | 27 |
| Multiple myeloma | Autologous HSCT | 56 | 21 |
| Leukemia | Allogeneic HSCT | 76 | 29 |

Abbreviations: AKI, acute kidney injury; HSCT, hematopoietic stem-cell transplantation.

**Supplementary Table 6.** Ninety-day HR of death by cancer and treatment.

| **Cancer** | **Treatment** | **90-day HR (95% CI)** |
| --- | --- | --- |
| Esophagus | Esophagus res | 2.2 (1.1-4.5) |
| Esophagus | Oxaliplatin | 6.3 (5.1-7.9) |
| Esophagus | Docetaxel | 6.1 (4.2-8.7) |
| Stomach | Gastrectomy | 3.9 (2.3-6.8) |
| Stomach | Oxaliplatin | 8.7 (7.1-11) |
| Stomach | Docetaxel | 9.5 (6.6-14) |
| Colon | Colectomy | 5.6 (4.9-6.4) |
| Colon | HIPEC | -^a^ |
| Colon | Oxaliplatin | 12.7 (11-15) |
| Colon | Bevacizumab | 9.0 (7.7-10) |
| Colon | Cetuximab | 9.6 (7.2-13) |
| Rectum | Ex of rectum | 5.4 (4.0-7.5) |
| Rectum | Oxaliplatin | 11.2 (8.5-15) |
| Rectum | Bevacizumab | 12.1 (9.4-16) |
| Rectum | Cetuximab | 15.8 (9.3-27) |
| Liver | Destruction of liver | 3.2 (1.0-11) |
| Liver | Liver res | 6.0 (2.0-18) |
| Liver | Cisplatin | 13.5 (7.1-26) |
| Liver | Oxaliplatin | 9.3 (5.6-16) |
| Liver | Doxorubicin | 4.9 (2.5-9.5) |
| Liver | TKI | 9.0 (6.6-12) |
| Pancreas | Ex of pancreas | 4.6 (2.5-8.5) |
| Pancreas | Oxaliplatin | 9.3 (7.9-11) |
| Pancreas | Gemcitabine | 7.6 (6.9-8.4) |
| Gall bladder | Cholecystectomy | 12.7 (1.6-104) |
| Gall bladder | Cisplatin | 8.3 (5.1-14) |
| Gall bladder | Oxaliplatin | 9.8 (6.7-14) |
| Lung | Lobectomy of lung | 3.8 (2.6-5.6) |
| Lung | Minor res of lung | 2.3 (1.1-4.7) |
| Lung | Cisplatin | 10.0 (7.6-13) |
| Lung | Carboplatin | 7.5 (7.1-8.0) |
| Lung | Bevacizumab | 8.5 (5.7-13) |
| Lung | ICI | 8.0 (7.1-9.1) |
| Lung | TKI | 6.9 (5.6-8.3) |
| Breast | Breast surgery | 15.0 (3.2-70) |
| Breast | CDK4/6i | 10.4 (5.9-18) |
| Breast | Trastuzumab | 46.7 (24-92) |
| Breast | Cyclophos | 38.0 (21-67) |
| Cervix | Total hysterectomy | -^a^ |
| Cervix | Cisplatin | 56.4 (23-136) |
| Cervix | Carboplatin | 3.4 (1.6-7.5) |
| Cervix | Bevacizumab | 4.7 (2.3-9.5) |
| Endometrium | Total hysterectomy | 7.2 (3.6-15) |
| Endometrium | Omentektomy | 4.9 (1.7-14) |
| Endometrium | Carboplatin | 23.6 (16-36) |
| Endometrium | Doxorubicin | 10.0 (6.6-15) |
| Ovary | Ex of ovary and tube | 7.4 (3.8-14) |
| Ovary | Omentektomy | 9.4 (4.8-18) |
| Ovary | Carboplatin | 15.1 (12-19) |
| Ovary | PARPi | 29.8 (16-54) |
| Ovary | Bevacizumab | 15.7 (12-21) |
| Ovary | Doxorubicin | 11.1 (8.9-14) |
| Kidney | Kidney res | 3.6 (1.0-13) |
| Kidney | Radical nephrectomy | 1.0 (0.6-1.7) |
| Kidney | ICI | 5.2 (3.4-7.9) |
| Kidney | TKI | 8.8 (6.9-11) |
| Urinary bladder | Cystectomy | 2.6 (1.8-3.8) |
| Urinary bladder | TURB | 3.6 (2.6-5.1) |
| Urinary bladder | Cisplatin | 5.9 (4.2-8.2) |
| Urinary bladder | Carboplatin | 7.2 (5.3-9.8) |
| Urinary bladder | ICI | 9.2 (6.6-13) |
| Prostate | Total ex of prostate | 4.8 (1.6-15) |
| Prostate | Docetaxel | 14.6 (12-18) |
| Testis | Orchiectomy | -^a^ |
| Testis | Cisplatin | 24.6 (5.9-103) |
| Brain | Brain surgery | 2.4 (1.4-4.0) |
| Brain | Bevacizumab | NA (NA-NA) |
| Melanoma | Dabra/Tram | 6.6 (3.6-12) |
| Melanoma | ICI | 10.5 (7.4-15) |
| Melanoma | Enco/bini | 3.1 (1.0-9.1) |
| Non-Hodgkin lymphoma | CHOP/CHOEP | 14.6 (11-19) |
| Non-Hodgkin lymphoma | Bendamustin | 16.8 (12-24) |
| Non-Hodgkin lymphoma | Autologous HSCT | 14.5 (5.3-40) |
| Hodgkin lymphoma | ABVD | -^a^ |
| Hodgkin lymphoma | Autologous HSCT | -^a^ |
| Hodgkin lymphoma | BEACOPP | -^a^ |
| Multiple myeloma | Bortezomib | 17.3 (12-24) |
| Multiple myeloma | Daratumumab | 35.9 (22-58) |
| Multiple myeloma | Carfilzomib | 11.3 (6.7-19) |
| Multiple myeloma | Autologous HSCT | 13.0 (3.1-54) |
| Leukemia | Allogeneic HSCT | 7.2 (1.5-35) |
| ALL | All drug | 3.3 (1.4-8.1) |
| AML | High dose chemo | 5.1 (3.3-8.0) |
| AML | Low dose chemo | 5.8 (4.5-7.5) |
| CLL | Cyclophos | 27.7 (5.6-138) |
| CLL | Bendamustin | 14.5 (4.4-48) |
| CLL | Ibrutinib | 79.6 (20-318) |
| CLL | Venetoclax | 25.9 (5.5-121) |
| CLL | All drugs | 9.2 (6.3-13) |
| CML | Imatinib | 58.2 (14-240) |
| CML | 2nd gen TKI | 78.2 (10-580) |
| CML | All drug | 22.6 (9.3-55) |
| Other leukemias | All drugs | 11.5 (8.5-16) |

^a^Cells are masked, so it is not possible to identify or back-calculate numbers less than 5.
Abbreviations: ABVD, adriamycin+bleomycin+vinblastine+dacarbazine; ALL, acute lymphatic leukemia; AML, acute myeloid leukemia; BEACOPP, bleomycin+etoposide+adriamycin+cyclophosphamide+vincristine+procarbazine+prednisone; CDK4/6i, Cyclin-dependent kinase 4/6 inhibitors; chemo, chemotherapy; CHOP/CHOEP, cyclophosphamide+hydroxydaunorubicin+vincristine+(etoposide)+prednisone ; CLL, chronic lymphatic leukemia; CML, chronic myeloid leukemia; cyclophos, cyclophosphamide; dabra/tram, dabrafenib/trametinib; enco/bini, encorafenib/binimetinib; ex, excision; gen, generation; HIPEC, hyperthermic intraperitoneal chemotherapy; HR, hazard ratio; HSCT, hematopoietic stem-cell transplantation; ICI, immune checkpoint inhibitors; PARPi, poly(ADP-ribose) polymerase inhibitors; res, resection; TKI, tyrosine kinase inhibitors. TURB, transurethral resection of bladder.
NB: patients could be included in multiple treatment groups.
