# Supplementary material for: Risk of and Mortality After Acute Kidney Injury Following Cancer Treatment: A Cohort Study
Source: Cancer Med. 2025 Feb 10;14(3):e70646. doi: 10.1002/cam4.70646 (PMC11808929; doi:10.1002/cam4.70646)
Supplement: Supplementary file 2 — Data S2 Supporting Information. [file CAM4-14-e70646-s002.docx]

**SUPPLEMENTARY MATERIAL**

**Content:
Supplementary Table 1.** Coding appendix.
**Supplementary Table 2**. Patients’ characteristics by cancer and treatment.
**Supplementary Table 3.** Distribution of stage and duration of AKI events after surgery.
**Supplementary Table 4.** Distribution of stage and duration of AKI events after anticancer drugs.
**Supplementary Table 5.** Distribution of stage and duration of AKI events after HSCT.
**Supplementary Table 6.** Ninety-day HR of death by cancer and treatment.

**Supplementary Table 1.** Coding appendix.

| **Condition** | **Source/coding system** | **Inclusion codes** |
| --- | --- | --- |
| **Cancer diagnosis** |  |  |
| Prostate | The patient registry/ ICD-10 | C61 |
| Urinary bladder | The patient registry/ ICD-10 | C67 |
| Kidney | The patient registry/ ICD-10 | C64 |
| Ovary | The patient registry/ ICD-10 | C56, C570, C571, C572, C573, C574 |
| Endometrium | The patient registry/ ICD-10 | C54, C55 |
| Cervix | The patient registry/ ICD-10 | C53 |
| Testis | The patient registry/ ICD-10 | C62 |
| Colon | The patient registry/ ICD-10 | C18, C19 |
| Rectum | The patient registry/ ICD-10 | C20 |
| Stomach | The patient registry/ ICD-10 | C16 |
| Esophagus | The patient registry/ ICD-10 | C15 |
| Liver | The patient registry/ ICD-10 | C22 |
| Gall bladder | The patient registry/ ICD-10 | C23, C24 |
| Pancreas | The patient registry/ ICD-10 | C25 |
| Breast | The patient registry/ ICD-10 | C50 |
| Lung | The patient registry/ ICD-10 | C33, C34 |
| Melanoma | The patient registry/ ICD-10 | C43 |
| Hodgkin lymphoma | The patient registry/ ICD-10 | C81 |
| Non-Hodgkin lymphoma | The patient registry/ ICD-10 | C82, C83, C84, C85, C86, C88 |
| ALL | The patient registry/ ICD-10 | C910 |
| CLL | The patient registry/ ICD-10 | C911 |
| AML | The patient registry/ ICD-10 | C920, C923, C924, C925, C926, C928 |
| CML | The patient registry/ ICD-10 | C921, C922 |
| Leukemia | The patient registry/ ICD-10 | C910, C911, C920, C921, C913, C914, C915, C916, C917, C918, C919, C922, C923, C924, C925, C926, C927, C928, C929, C93, C94, C95 |
| Other leukemias | The patient registry/ ICD-10 | C913, C914, C915 C916, DC917, C918, C919, C922, C927, C929, C93, C94, C95 |
| Multiple myeloma | The patient registry/ ICD-10 | C900 |
| Brain | The patient registry/ ICD-10 | C71 |
| **Surgical procedures** |  |  |
| Cholecystectomy | The patient registry/NCSP | KJKA2 |
| Colectomy | The patient registry/NCSP | KJFB, KJFH |
| Cystectomy | The patient registry/NCSP | KKCC |
| Destruction of liver | The patient registry/NCSP | KJJA43 |
| Res of esophagus | The patient registry/NCSP | KJCC |
| Salpingo-oophorectomy | The patient registry/NCSP | KLAF |
| Ex of pancreas | The patient registry/NCSP | KJLC |
| Ex of rectum | The patient registry/NCSP | KJGB |
| Gastrectomy | The patient registry/NCSP | KJDC, KJDD, KJDE |
| Partial res of kidney | The patient registry/NCSP | KKAD |
| Lobectomy of lung | The patient registry/NCSP | KGDC |
| Minor res of lung | The patient registry/NCSP | KGDB |
| Radical nephrectomy | The patient registry/NCSP | KKAC |
| Orchiectomy | The patient registry/NCSP | KKFC0, KKFC1 |
| Breast surgery | The patient registry/NCSP | KHAC25, KHAB40, KHAC20 |
| Brain surgery | The patient registry/NCSP | KAAG, KAAB |
| Radical prostatectomy | The patient registry/NCSP | KKEC |
| Total hysterectomy | The patient registry/NCSP | KLCD |
| TURB | The patient registry/NCSP | KKCD32 |
| HIPEC | The patient registry/NCSP | KJAQ1 |
| Omentectomy | The patient registry/NCSP | KJAL3 |
| Res of liver | The patient registry/NCSP | KJJB |
| **Anticancer drugs** |  |  |
| Cisplatin | The patient registry/Procedure | BWHA107, BWHA126, BWHA128, BWHA132, BWHA133, BWHA140, BWHA184, BWHA185, BWHA201, BWHA206, BWHA207, BWHA209, BWHA214, BWHA224, BWHA225, BWHA226, BWHA240, BWHA251, BWHA261 |
| Carboplatin | The patient registry/Procedure | BWHA109, BWHA112, BWHA127, BWHA129, BWHA130, BWHA157, BWHA203, BWHA214, BWHA224, BWHA238, BWHA242, BWHA252 |
| Oxaliplatin | The patient registry/Procedure | BWHA108, BWHA222, BWHA223, BWHA231, BWHA234, BWHA253, BWHA254 |
| Gemcitabine | The patient registry/Procedure | BWHA114, BWHA128, BWHA129, BWHA170, BWHA206, BWHA211, BWHA235, BWHA236, BWHA238, BWHA253, BWHA259 |
| PARPi | The patient registry/Procedure | BWHA433, BWHA437 |
| CDK4/6i | The patient registry/Procedure | BWHA442, BWHA444 |
| Bevacizumab | The patient registry/Procedure | BOHJ19B1 |
| Trastuzumab | The patient registry/Procedure | BOHJ13 |
| Cyclophos | The patient registry/Procedure | BWHA105, BWHA117, BWHA118, BWHA119, BWHA134, BWHA139, BWHA144, BWHA156, BWHA160, BWHA164, BWHA165, BWHA166, BWHA174, BWHA175, BWHA176, BWHA218, BWHA241, BWHA247, BWHA311, BWHA312 |
| CHOP/CHOEP | The patient registry/Procedure | BWHA165, BWHA119 |
| Bendamustin | The patient registry/Procedure | BWHA177 |
| ABVD | The patient registry/Procedure | BWHA167 |
| Dabra/tram | The patient registry/Procedure | BWHA419, BWHA420 |
| Bortezomib | The patient registry/Procedure | BWHA402 |
| Daratumumab | The patient registry/Procedure | BOHJ19H8 |
| Carfilzomib | The patient registry/Procedure | BWHA432 |
| Docetaxel | The patient registry/Procedure | BWHA208, BWHA209, BWHA210, BWHA211, BWHA247, BWHA252 |
| Doxorubicin | The patient registry/Procedure | BWHA102, BWHA119, BWHA144, BWHA146, BWHA165, BWHA170, BWHA183, BWHA184, BWHA185, BWHA216, BWHA232, BWHA237, BWHA258, BWHA259, BWHA260 |
| Cetuximab | The patient registry/Procedure | BOHJ17 |
| ICI | The patient registry/Procedure | BOHJ19H2, BOHJ19H7, BOHJ19J, BOHJ19D |
| TKI | The patient registry/Procedure | BWHA401, BWHA404, BWHA407, BWHA406, BWHA410, BWHA413, BWHA426, BWHA424, BWHA422, BWHA420, BWHA417, BWHA405, BWHA440, BWHA434, BWHA414, BWHP120, BWHA447, BWHA448, BWHA44, BWHA46, BWHA47, BWHA427 |
| Enco/bini | The patient registry/Procedure | BWHA446, BWHA447 |
| All drugs | The patient registry/Procedure | BWHA, BWHB1, BWHB2, BWHB3, BWHB8, BOHJ1 |
| High dose chemo | The patient registry/Procedure | BWHA30 |
| Low dose chemo | The patient registry/Procedure | BWHA158, BWHA256 |
| Imatinib | The patient registry/Procedure | BWHA401 |
| 2nd gen TKI | The patient registry/Procedure | BWHA411, BWHA409, BWHA425 |
| Ibrutinib | The patient registry/Procedure | BWHA427 |
| Venetoclax | The patient registry/Procedure | BWHA438 |
| BEACOPP | The patient registry/Procedure | BWHA138 |
| **HSCT** |  |  |
| Autologous HSCT | The patient registry/Procedure | BOQE1, BOQE2, BOQF0 |
| Allogeneic HSCT | The patient registry/Procedure | BOQE3, BOQE4, BOQE5, BOQE6, BOQF1, BOQF2 |
| **Dialysis** |  |  |
| Chronic dialysis | The patient registry/Procedure | BJFD2 |
| Acute dialysis | The patient registry/Procedure | BJFD0 |
| **Charlson comorbidity index score** |  |  |
| Myocardial infarction | The patient registry/ICD-10 | I21, I22, I23 |
| Congestive heart failure | The patient registry/ICD-10 | I50, I110, I130, I132 |
| Peripheral vascular disease | The patient registry/ICD-10 | I70, I71, I72, I73, I74, I77 |
| Cerebrovascular disease | The patient registry/ICD-10 | I60-I69, G45, G46 |
| Dementia | The patient registry/ICD-10 | F00-F03, F05.1, G30 |
| Chronic pulmonary disease | The patient registry/ICD-10 | J40-J47, J60-J67, J68.4, J701,  J703, J841, J920, J961, J982,  J983 |
| Connective tissue disorder | The patient registry/ICD-10 | M05, M06, M08, M09, M30, M31, M32, M33, M34, M35, M36, D86 |
| Ulcer disease | The patient registry/ICD-10 | K22.1, K25-K28 |
| Mild liver disease | The patient registry/ICD-10 | B18, K700-K703, K709, K71,  K73, K74, K760 |
| Diabetes mellitus | The patient registry/ICD-10 | E100, E101, E109, E110, E111, E119 |
| Hemiplegia | The patient registry/ICD-10 | G81, G82 |
| Moderate to severe renal  disease | The patient registry/ICD-10 | I12, I13, N00-N05, N07, N11, N14, N17-N19, Q61 |
| Diabetes with end organ damage | The patient registry/ICD-10 | E102-E108, E112-E118 |
| Moderate to severe liver disease | The patient registry/ICD-10 | B150, B160, B162, B190, K704, K72, K766, I85 |
| AIDS | The patient registry/ICD-10 | B21-B24 |
| **Metastasis** |  |  |
| Distant metastasis | The patient registry/ICD-10 | C78, C79, CxxxM |
|  | The pathology registry/SNOMED | M8xxx6, M9xxx6 |

Abbreviations: ABVD, adriamycin+bleomycin+vinblastine+dacarbazine; AIDS, acquired immunodeficiency syndrome; ALL, acute lymphatic leukemia; AML, acute myeloid leukemia; BEACOPP, bleomycin+etoposide+adriamycin+cyclophosphamide+vincristine+procarbazine+prednisone; CDK4/6i, Cyclin-dependent kinase 4/6 inhibitors; chemo, chemotherapy; CHOP/CHOEP, cyclophosphamide+hydroxydaunorubicin+vincristine+(etoposide)+prednisone ; CLL, chronic lymphatic leukemia; CML, chronic myeloid leukemia; cyclophos, cyclophosphamide; dabra/tram, dabrafenib/trametinib; enco/bini, encorafenib/binimetinib; ex, excision; gen, generation; HIPEC, hyperthermic intraperitoneal chemotherapy; HSCT, hematopoietic stem-cell transplantation; ICD, International Classification of Diseases ; ICI, immune checkpoint inhibitors; NCSP, Nordic Medico-Statistical Committee Classification of Surgical Procedures; PARPi, poly(ADP-ribose) polymerase inhibitors; res, resection; SNOMED, Systematized Nomenclature of Medicine; TKI, tyrosine kinase inhibitors. TURB, transurethral resection of bladder.
NB: patients could be included in multiple treatment groups.

**Supplementary Table 2.** Patients’ characteristics by cancer and treatment.

| **Cancer** | **Treatment** | **N patients** | **Age, median (Q1-Q3)** | **Female sex, %** | **eGFR, median (Q1-Q3)** | **Low/moderate/high CCIs, %** | **Distant metastasis, %** |
| --- | --- | --- | --- | --- | --- | --- | --- |
| Esophagus | Esophagus res | 1376 | 67 (61 to 73) | 22 | 86 (72 to 94) | 58/35/7 | 48 |
| Esophagus | Oxaliplatin | 1479 | 67 (61 to 73) | 20 | 86 (74 to 94) | 62/33/6 | 42 |
| Esophagus | Docetaxel | 666 | 66 (60 to 72) | 20 | 86 (74 to 94) | 61/34/5 | 29 |
| Stomach | Gastrectomy | 1742 | 68 (60 to 75) | 36 | 83 (70 to 92) | 50/42/8 | 44 |
| Stomach | Oxaliplatin | 2276 | 67 (59 to 73) | 27 | 85 (72 to 94) | 60/35/5 | 43 |
| Stomach | Docetaxel | 1147 | 65 (57 to 71) | 26 | 86 (74 to 94) | 62/34/5 | 31 |
| Colon | Colectomy | 22305 | 72 (64 to 79) | 50 | 80 (65 to 89) | 59/33/8 | 12 |
| Colon | HIPEC | 420 | 62 (53 to 69) | 60 | 89 (81 to 97) | 74/22/4 | 93 |
| Colon | Oxaliplatin | 6622 | 65 (58 to 71) | 46 | 86 (75 to 94) | 72/24/3 | 40 |
| Colon | Bevacizumab | 3081 | 68 (60 to 75) | 46 | 84 (71 to 92) | 70/25/4 | 81 |
| Colon | Cetuximab | 905 | 66 (59 to 72) | 43 | 86 (74 to 94) | 70/26/5 | 78 |
| Rectum | Ex of rectum | 9242 | 69 (61 to 75) | 37 | 84 (71 to 93) | 68/28/5 | 10 |
| Rectum | Oxaliplatin | 2622 | 64 (56 to 69) | 36 | 89 (78 to 97) | 76/21/3 | 44 |
| Rectum | Bevacizumab | 1392 | 66 (59 to 73) | 33 | 87 (75 to 95) | 69/27/4 | 77 |
| Rectum | Cetuximab | 421 | 63 (56 to 69) | 33 | 89 (78 to 97) | 74/22/4 | 70 |
| Liver | Destruction of liver | 578 | 67 (61 to 73) | 22 | 88 (72 to 97) | 25/29/46 | 7 |
| Liver | Liver res | 548 | 68 (60 to 74) | 33 | 88 (75 to 97) | 44/33/23 | 15 |
| Liver | Cisplatin | 323 | 66 (58 to 73) | 48 | 90 (77 to 97) | 58/33/9 | 52 |
| Liver | Oxaliplatin | 300 | 66 (58 to 72) | 50 | 87 (74 to 96) | 60/31/9 | 59 |
| Liver | Doxorubicin | 278 | 68 (61 to 74) | 19 | 89 (75 to 99) | 31/24/45 | 9 |
| Liver | TKI | 650 | 68 (62 to 74) | 18 | 87 (73 to 97) | 39/26/35 | 29 |
| Pancreas | Ex of pancreas | 1795 | 68 (60 to 74) | 45 | 89 (77 to 97) | 62/32/6 | 41 |
| Pancreas | Oxaliplatin | 2209 | 65 (58 to 70) | 44 | 92 (83 to 99) | 69/26/5 | 59 |
| Pancreas | Gemcitabine | 4528 | 69 (63 to 75) | 47 | 88 (77 to 96) | 62/31/7 | 58 |
| Gall bladder | Cholecystectomy | 209 | 69 (62 to 75) | 57 | 83 (67 to 93) | 54/33/12 | 33 |
| Gall bladder | Cisplatin | 415 | 66 (58 to 72) | 62 | 90 (77 to 98) | 70/24/6 | 60 |
| Gall bladder | Oxaliplatin | 528 | 67 (60 to 73) | 53 | 88 (75 to 96) | 67/26/8 | 62 |
| Lung | Lobectomy of lung | 7929 | 69 (63 to 75) | 54 | 85 (71 to 93) | 49/41/9 | 23 |
| Lung | Minor res of lung | 1903 | 70 (64 to 76) | 59 | 83 (67 to 92) | 37/50/13 | 21 |
| Lung | Cisplatin | 2915 | 64 (57 to 69) | 54 | 91 (81 to 98) | 63/33/4 | 64 |
| Lung | Carboplatin | 16079 | 69 (62 to 74) | 49 | 87 (75 to 95) | 51/41/8 | 76 |
| Lung | Bevacizumab | 598 | 65 (59 to 71) | 58 | 90 (79 to 98) | 63/34/4 | 85 |
| Lung | ICI | 5308 | 68 (62 to 74) | 51 | 88 (76 to 95) | 54/39/7 | 85 |
| Lung | TKI | 2182 | 68 (61 to 75) | 60 | 86 (74 to 95) | 62/32/6 | 84 |
| Breast | Breast surgery | 35884 | 64 (54 to 73) | 99 | 86 (74 to 95) | 72/25/3 | 38 |
| Breast | CDK4/6i | 1181 | 65 (53 to 74) | 99 | 84 (71 to 95) | 72/23/5 | 87 |
| Breast | Trastuzumab | 5490 | 59 (49 to 68) | 99 | 90 (78 to 99) | 77/21/2 | 39 |
| Breast | Cyclophos | 15570 | 54 (47 to 63) | 100 | 92 (81 to 101) | 80/18/2 | 41 |
| Cervix | Total hysterectomy | 1203 | 46 (39 to 59) | 100 | 95 (83 to 107) | 77/21/2 | 11 |
| Cervix | Cisplatin | 1083 | 51 (41 to 62) | 100 | 94 (83 to 105) | 78/20/2 | 19 |
| Cervix | Carboplatin | 259 | 58 (45 to 68) | 100 | 85 (69 to 97) | 67/30/3 | 54 |
| Cervix | Bevacizumab | 230 | 52 (43 to 63) | 100 | 89 (76 to 102) | 72/25/3 | 63 |
| Endometrium | Total hysterectomy | 6484 | 69 (61 to 76) | 100 | 80 (66 to 91) | 69/27/5 | 14 |
| Endometrium | Omentektomy | 1092 | 70 (63 to 75) | 100 | 82 (69 to 91) | 71/26/3 | 42 |
| Endometrium | Carboplatin | 1454 | 69 (62 to 75) | 100 | 82 (68 to 91) | 70/27/3 | 70 |
| Endometrium | Doxorubicin | 521 | 67 (60 to 72) | 100 | 84 (70 to 93) | 68/28/4 | 79 |
| Ovary | Ex of ovary and tube | 3358 | 65 (56 to 73) | >99 | 85 (72 to 95) | 73/24/3 | 70 |
| Ovary | Omentektomy | 3372 | 65 (56 to 73) | >99 | 86 (73 to 95) | 74/24/2 | 70 |
| Ovary | Carboplatin | 3717 | 68 (58 to 75) | 100 | 84 (70 to 93) | 71/25/3 | 68 |
| Ovary | PARPi | 668 | 63 (55 to 71) | 100 | 87 (75 to 96) | 73/25/2 | 93 |
| Ovary | Bevacizumab | 1338 | 66 (58 to 73) | 100 | 85 (73 to 93) | 73/25/2 | 89 |
| Ovary | Doxorubicin | 1623 | 66 (58 to 72) | 100 | 84 (71 to 93) | 71/26/3 | 89 |
| Kidney | Kidney res | 1997 | 63 (55 to 70) | 32 | 86 (71 to 96) | 59/33/8 | 2 |
| Kidney | Radical nephrectomy | 3396 | 66 (58 to 73) | 33 | 81 (66 to 93) | 62/30/8 | 14 |
| Kidney | ICI | 979 | 64 (57 to 70) | 26 | 82 (67 to 94) | 65/29/6 | 87 |
| Kidney | TKI | 1430 | 65 (58 to 72) | 27 | 80 (64 to 92) | 61/30/9 | 83 |
| Urinary bladder | Cystectomy | 3317 | 70 (63 to 75) | 26 | 79 (64 to 90) | 63/32/5 | 20 |
| Urinary bladder | TURB | 5858 | 74 (67 to 80) | 24 | 75 (58 to 87) | 52/37/11 | 3 |
| Urinary bladder | Cisplatin | 1480 | 66 (60 to 71) | 25 | 85 (70 to 93) | 70/27/3 | 28 |
| Urinary bladder | Carboplatin | 738 | 72 (66 to 76) | 26 | 71 (57 to 87) | 54/38/8 | 59 |
| Urinary bladder | ICI | 719 | 70 (63 to 75) | 24 | 78 (61 to 90) | 57/35/8 | 73 |
| Prostate | Total ex of prostate | 12053 | 66 (61 to 70) | 0 | 84 (74 to 91) | 76/22/2 | 9 |
| Prostate | Docetaxel | 4334 | 70 (65 to 75) | 0 | 82 (70 to 90) | 66/29/5 | 72 |
| Testis | Orchiectomy | 1920 | 37 (29 to 47) | 0 | 103 (91 to 112) | 87/12/1 | 5 |
| Testis | Cisplatin | 788 | 35 (28 to 45) | <1 | 105 (93 to 114) | 87/*/* | 51 |
| Brain | Brain surgery | 4016 | 63 (54 to 71) | 40 | 85 (73 to 95) | 68/28/4 | 6 |
| Brain | Bevacizumab | 1054 | 57 (48 to 66) | 38 | 89 (78 to 98) | 73/25/2 | 4 |
| Melanoma | Dabra/Tram | 367 | 65 (53 to 74) | 39 | 85 (74 to 95) | 58/35/7 | 96 |
| Melanoma | ICI | 1674 | 67 (57 to 75) | 40 | 84 (71 to 93) | 66/30/5 | 95 |
| Melanoma | Enco/bini | 160 | 63 (54 to 71) | 39 | 86 (72 to 94) | 57/38/5 | 94 |
| Non-Hodgkin lymphoma | CHOP/CHOEP | 3676 | 68 (59 to 76) | 42 | 83 (68 to 93) | 63/32/6 | NA |
| Non-Hodgkin lymphoma | Bendamustin | 1896 | 69 (61 to 75) | 43 | 81 (67 to 91) | 60/34/7 | NA |
| Non-Hodgkin lymphoma | Autologous HSCT | 586 | 58 (51 to 64) | 32 | 91 (80 to 100) | 73/23/4 | NA |
| Hodgkin lymphoma | ABVD | 623 | 45 (27 to 63) | 41 | 100 (87 to 115) | 72/24/4 | NA |
| Hodgkin lymphoma | Autologous HSCT | 63 | 43 (27 to 59) | 29 | 104 (90 to 118) | 70/*/* | NA |
| Hodgkin lymphoma | BEACOPP | 167 | 28 (22 to 37) | 41 | 116 (106 to 126) | 84/*/* | NA |
| Multiple myeloma | Bortezomib | 2925 | 70 (62 to 76) | 43 | 75 (58 to 88) | 61/31/8 | NA |
| Multiple myeloma | Daratumumab | 1452 | 71 (63 to 76) | 43 | 75 (59 to 88) | 53/36/11 | NA |
| Multiple myeloma | Carfilzomib | 536 | 64 (57 to 71) | 42 | 78 (64 to 93) | 62/30/8 | NA |
| Multiple myeloma | Autologous HSCT | 1056 | 61 (55 to 65) | 42 | 83 (66 to 95) | 71/26/3 | NA |
| Leukemia | Allogeneic HSCT | 657 | 56 (43 to 64) | 42 | 90 (77 to 99) | 73/25/2 | NA |
| ALL | All drug | 305 | 46 (28 to 65) | 39 | 86 (68 to 99) | 74/22/4 | NA |
| AML | High dose chemo | 729 | 60 (49 to 68) | 44 | 88 (74 to 98) | 75/22/3 | NA |
| AML | Low dose chemo | 808 | 72 (63 to 78) | 39 | 79 (65 to 89) | 55/36/8 | NA |
| CLL | Cyclophos | 208 | 63 (56 to 69) | 30 | 82 (72 to 92) | 68/27/5 | NA |
| CLL | Bendamustin | 400 | 68 (63 to 74) | 28 | 78 (65 to 88) | 66/28/6 | NA |
| CLL | Ibrutinib | 293 | 68 (60 to 75) | 29 | 77 (64 to 88) | 63/28/9 | NA |
| CLL | Venetoclax | 311 | 66 (58 to 73) | 27 | 81 (68 to 91) | 58/36/6 | NA |
| CLL | All drugs | 1646 | 69 (62 to 76) | 34 | 78 (64 to 89) | 58/35/7 | NA |
| CML | Imatinib | 598 | 59 (46 to 70) | 45 | 84 (72 to 97) | 68/25/7 | NA |
| CML | 2nd gen TKI | 385 | 56 (44 to 67) | 44 | 86 (72 to 99) | 67/26/7 | NA |
| CML | All drug | 761 | 61 (47 to 72) | 44 | 84 (70 to 97) | 68/25/7 | NA |
| Other leukemias | All drugs | 1214 | 70 (61 to 78) | 37 | 78 (61 to 89) | 54/37/9 | NA |

*Cells are masked, so it is not possible to identify or back-calculate numbers less than 5.
Abbreviations: ABVD, adriamycin+bleomycin+vinblastine+dacarbazine; ALL, acute lymphatic leukemia; AML, acute myeloid leukemia; BEACOPP, bleomycin+etoposide+adriamycin+cyclophosphamide+vincristine+procarbazine+prednisone; CCIs, Charlson Comorbidity Index score; CDK4/6i, Cyclin-dependent kinase 4/6 inhibitors; chemo, chemotherapy; CHOP/CHOEP, cyclophosphamide+hydroxydaunorubicin+vincristine+(etoposide)+prednisone ; CLL, chronic lymphatic leukemia; CML, chronic myeloid leukemia; cyclophos, cyclophosphamide; dabra/tram, dabrafenib/trametinib; eGFR, estimated glomerular filtration rate; enco/bini, encorafenib/binimetinib; ex, excision; gen, generation; HIPEC, hyperthermic intraperitoneal chemotherapy; HSCT, hematopoietic stem-cell transplantation; ICI, immune checkpoint inhibitors; PARPi, poly(ADP-ribose) polymerase inhibitors; Q1, first quartile; Q3 third quartile; res, resection; TKI, tyrosine kinase inhibitors. TURB, transurethral resection of bladder.
NB: patients could be included in multiple treatment groups.

**Supplementary Table 3.** Distribution of stage and duration of AKI events after surgery.

| **Cancer** | **Treatment** | **Persistent AKI, %** | **Stage 2-3 AKI, %** |
| --- | --- | --- | --- |
| Esophagus | Esophagus res | 35 | 30 |
| Stomach | Gastrectomy | 38 | 30 |
| Colon | Colectomy | 35 | 36 |
| Colon | HIPEC | * | 33 |
| Rectum | Ex of rectum | 39 | 37 |
| Liver | Destruction of liver | 40 | 27 |
| Liver | Liver res | 47 | 32 |
| Pancreas | Ex of pancreas | 33 | 32 |
| Gall bladder | Cholecystectomy | * | 37 |
| Lung | Lobectomy of lung | 37 | 29 |
| Lung | Minor res of lung | 44 | 29 |
| Breast | Breast surgery | 15 | 29 |
| Cervix | Total hysterectomy | 24 | 24 |
| Endometrium | Total hysterectomy | 22 | 31 |
| Endometrium | Omentectomy | 18 | 33 |
| Ovary | Ex of ovary and tube | 29 | 28 |
| Ovary | Omentectomy | 28 | 27 |
| Kidney | Kidney res | 54 | 22 |
| Kidney | Radical nephrectomy | 87 | 20 |
| Urinary bladder | Cystectomy | 51 | 43 |
| Urinary bladder | TURB | 51 | 30 |
| Prostate | Total ex of prostate | 22 | 27 |
| Testis | Orchiectomy | * | * |
| Brain | Brain surgery | 36 | 21 |

*Cells are masked, so it is not possible to identify or back-calculate numbers less than 5.
Abbreviations: AKI, acute kidney injury; ex, excision; HIPEC, hyperthermic intraperitoneal chemotherapy; res, resection; TURB, transurethral resection of bladder.
NB: patients could be included in multiple treatment groups.

**Supplementary Table 4.** Distribution of stage and duration of AKI events after anticancer drugs.

| **Cancer** | **Treatment** | **Persistent AKI, %** | **Stage 2-3 AKI, %** |
| --- | --- | --- | --- |
| Esophagus | Oxaliplatin | 35 | 30 |
| Esophagus | Docetaxel | 32 | 25 |
| Stomach | Oxaliplatin | 40 | 31 |
| Stomach | Docetaxel | 37 | 30 |
| Colon | Oxaliplatin | 38 | 33 |
| Colon | Bevacizumab | 37 | 34 |
| Colon | Cetuximab | 32 | 33 |
| Rectum | Oxaliplatin | 40 | 35 |
| Rectum | Bevacizumab | 39 | 36 |
| Rectum | Cetuximab | 46 | 41 |
| Liver | Cisplatin | 33 | 31 |
| Liver | Oxaliplatin | 43 | 38 |
| Liver | Doxorubicin | 44 | 34 |
| Liver | TKI | 43 | 39 |
| Pancreas | Oxaliplatin | 32 | 34 |
| Pancreas | Gemcitabine | 34 | 34 |
| Gall bladder | Cisplatin | 38 | 33 |
| Gall bladder | Oxaliplatin | 36 | 42 |
| Lung | Cisplatin | 43 | 26 |
| Lung | Carboplatin | 33 | 28 |
| Lung | Bevacizumab | 31 | 27 |
| Lung | ICI | 37 | 26 |
| Lung | TKI | 33 | 28 |
| Breast | CDK4/6i | 56 | 23 |
| Breast | Trastuzumab | 40 | 29 |
| Breast | Cyclophos | 33 | 23 |
| Cervix | Cisplatin | 49 | 29 |
| Cervix | Carboplatin | 53 | 44 |
| Cervix | Bevacizumab | 50 | 33 |
| Endometrium | Carboplatin | 43 | 32 |
| Endometrium | Doxorubicin | 46 | 43 |
| Ovary | Carboplatin | 42 | 25 |
| Ovary | PARPi | 40 | 30 |
| Ovary | Bevacizumab | 42 | 24 |
| Ovary | Doxorubicin | 45 | 29 |
| Kidney | ICI | 57 | 38 |
| Kidney | TKI | 45 | 33 |
| Urinary bladder | Cisplatin | 57 | 35 |
| Urinary bladder | Carboplatin | 51 | 36 |
| Urinary bladder | ICI | 59 | 35 |
| Prostate | Docetaxel | 50 | 32 |
| Testis | Cisplatin | 48 | 30 |
| Brain | Bevacizumab | 17 | 21 |
| Melanoma | Dabra/Tram | 38 | 27 |
| Melanoma | ICI | 53 | 37 |
| Melanoma | Enco/bini | 35 | 29 |
| Non-Hodgkin lymphoma | CHOP/CHOEP | 49 | 23 |
| Non-Hodgkin lymphoma | Bendamustin | 42 | 23 |
| Hodgkin lymphoma | ABVD | 41 | 24 |
| Hodgkin lymphoma | BEACOPP | 31 | 9 |
| Multiple myeloma | Bortezomib | 50 | 31 |
| Multiple myeloma | Daratumumab | 44 | 28 |
| Multiple myeloma | Carfilzomib | 50 | 32 |
| ALL | All drug | 45 | 26 |
| AML | High dose chemo | 56 | 25 |
| AML | Low dose chemo | 49 | 21 |
| CLL | Cyclophos | 45 | 20 |
| CLL | Bendamustin | 50 | 30 |
| CLL | Ibrutinib | 42 | 28 |
| CLL | Venetoclax | 38 | 19 |
| CLL | All drugs | 45 | 32 |
| CML | Imatinib | 51 | 24 |
| CML | 2nd gen TKI | 54 | 20 |
| CML | All drugs | 51 | 22 |
| Other leukemias | All drugs | 51 | 29 |

Abbreviations: ABVD, adriamycin+bleomycin+vinblastine+dacarbazine; AKI, acute kidney injury; BEACOPP, bleomycin+etoposide+adriamycin+cyclophosphamide+vincristine+procarbazine+prednisone; ALL, acute lymphatic leukemia; AML, acute myeloid leukemia; CDK4/6i, Cyclin-dependent kinase 4/6 inhibitors; chemo, chemotherapy; CHOP/CHOEP, cyclophosphamide+hydroxydaunorubicin+vincristine+(etoposide)+prednisone ; CLL, chronic lymphatic leukemia; CML, chronic myeloid leukemia; cyclophos, cyclophosphamide; dabra/tram, dabrafenib/trametinib; enco/bini, encorafenib/binimetinib; gen, generation; ICI, immune checkpoint inhibitors; PARPi, poly(ADP-ribose) polymerase inhibitors; TKI, tyrosine kinase inhibitors.
NB: patients could be included in multiple treatment groups.

**Supplementary Table 5.** Distribution of stage and duration of AKI events after HSCT.

| **Cancer** | **Treatment** | **Persistent AKI, %** | **Stage 2-3 AKI, %** |
| --- | --- | --- | --- |
| Non-Hodgkin lymphoma | Autologous HSCT | 55 | 21 |
| Hodgkin lymphoma | Autologous HSCT | 67 | 29 |
| Multiple myeloma | Autologous HSCT | 56 | 21 |
| Leukemia | Allogeneic HSCT | 76 | 29 |

Abbreviations: AKI, acute kidney injury; HSCT, hematopoietic stem-cell transplantation.

**Supplementary Table 6.** Ninety-day HR of death by cancer and treatment.

| **Cancer** | **Treatment** | **90-day HR (95% CI)** |
| --- | --- | --- |
| Esophagus | Esophagus res | 2.2 (1.0 to 4.8) |
| Esophagus | Oxaliplatin | 6.6 (5.3 to 8.3) |
| Esophagus | Docetaxel | 6.5 (4.4 to 9.6) |
| Stomach | Gastrectomy | 3.7 (2.1 to 6.5) |
| Stomach | Oxaliplatin | 8.2 (6.7 to 10) |
| Stomach | Docetaxel | 10.0 (6.9 to 14) |
| Colon | Colectomy | 5.5 (4.8 to 6.4) |
| Colon | HIPEC | 3.1 (0.5 to 20) |
| Colon | Oxaliplatin | 12.4 (11 to 14) |
| Colon | Bevacizumab | 9.4 (8.1 to 11) |
| Colon | Cetuximab | 9.2 (6.9 to 12) |
| Rectum | Ex of rectum | 6.2 (4.5 to 8.4) |
| Rectum | Oxaliplatin | 11.5 (8.7 to 15) |
| Rectum | Bevacizumab | 15.1 (12 to 19) |
| Rectum | Cetuximab | 12.5 (7.6 to 21) |
| Liver | Destruction of liver | 2.5 (0.9 to 7.0) |
| Liver | Liver res | 5.6 (2.3 to 14) |
| Liver | Cisplatin | 10.6 (5.8 to 19) |
| Liver | Oxaliplatin | 9.8 (5.8 to 17) |
| Liver | Doxorubicin | 4.7 (2.5 to 8.9) |
| Liver | TKI | 7.8 (5.7 to 11) |
| Pancreas | Ex of pancreas | 3.7 (2.0 to 6.6) |
| Pancreas | Oxaliplatin | 9.7 (8.2 to 12) |
| Pancreas | Gemcitabine | 7.4 (6.7 to 8.1) |
| Gall bladder | Cholecystectomy | 12.7 (1.6 to 104) |
| Gall bladder | Cisplatin | 8.7 (5.5 to 14) |
| Gall bladder | Oxaliplatin | 9.2 (6.3 to 14) |
| Lung | Lobectomy of lung | 3.3 (2.4 to 4.6) |
| Lung | Minor res of lung | 2.8 (1.4 to 5.6) |
| Lung | Cisplatin | 9.9 (7.7 to 13) |
| Lung | Carboplatin | 7.4 (7.0 to 7.9) |
| Lung | Bevacizumab | 6.3 (4.4 to 9.0) |
| Lung | ICI | 7.9 (6.9 to 8.9) |
| Lung | TKI | 6.6 (5.5 to 8.0) |
| Breast | Breast surgery | 7.5 (2.1 to 27) |
| Breast | CDK4/6i | 9.7 (5.6 to 17) |
| Breast | Trastuzumab | 38.2 (19 to 77) |
| Breast | Cyclophos | 32.5 (19 to 56) |
| Cervix | Total hysterectomy | 3.1 (0.2 to 49) |
| Cervix | Cisplatin | 46.5 (18 to 118) |
| Cervix | Carboplatin | 4.0 (1.8 to 8.5) |
| Cervix | Bevacizumab | 6.2 (3.0 to 13) |
| Endometrium | Total hysterectomy | 6.1 (2.9 to 13) |
| Endometrium | Omentektomy | 5.4 (1.8 to 16) |
| Endometrium | Carboplatin | 18.0 (13 to 26) |
| Endometrium | Doxorubicin | 9.5 (6.4 to 14) |
| Ovary | Ex of ovary and tube | 5.2 (2.6 to 10) |
| Ovary | Omentektomy | 8.9 (4.6 to 17) |
| Ovary | Carboplatin | 15.2 (12 to 19) |
| Ovary | PARPi | 37.3 (15 to 92) |
| Ovary | Bevacizumab | 19.1 (14 to 26) |
| Ovary | Doxorubicin | 11.9 (9.5 to 15) |
| Kidney | Kidney res | 5.7 (1.5 to 22) |
| Kidney | Radical nephrectomy | 1.1 (0.6 to 1.9) |
| Kidney | ICI | 6.8 (4.5 to 10) |
| Kidney | TKI | 9.0 (7.1 to 12) |
| Urinary bladder | Cystectomy | 2.4 (1.6 to 3.4) |
| Urinary bladder | TURB | 3.2 (2.2 to 4.5) |
| Urinary bladder | Cisplatin | 5.4 (3.9 to 7.5) |
| Urinary bladder | Carboplatin | 7.7 (5.6 to 11) |
| Urinary bladder | ICI | 9.8 (7.1 to 14) |
| Prostate | Total ex of prostate | 6.7 (2.5 to 18) |
| Prostate | Docetaxel | 16.8 (14 to 21) |
| Testis | Orchiectomy | * |
| Testis | Cisplatin | 19.0 (3.6 to 102) |
| Brain | Brain surgery | 2.3 (1.4 to 3.9) |
| Brain | Bevacizumab | 4.2 (2.6 to 6.8) |
| Melanoma | Dabra/Tram | 6.2 (3.5 to 11) |
| Melanoma | ICI | 9.8 (7.0 to 14) |
| Melanoma | Enco/bini | 3.2 (1.1 to 9.4) |
| Non-Hodgkin lymphoma | CHOP/CHOEP | 13.9 (11 to 18) |
| Non-Hodgkin lymphoma | Bendamustin | 18.4 (13 to 27) |
| Non-Hodgkin lymphoma | Autologous HSCT | 16.5 (7.0 to 39) |
| Hodgkin lymphoma | ABVD | 60.0 (8.2 to 437) |
| Hodgkin lymphoma | Autologous HSCT | * |
| Hodgkin lymphoma | BEACOPP | * |
| Multiple myeloma | Bortezomib | 18.3 (13 to 25) |
| Multiple myeloma | Daratumumab | 26.9 (16 to 45) |
| Multiple myeloma | Carfilzomib | 11.9 (7.2 to 20) |
| Multiple myeloma | Autologous HSCT | 30.9 (7.6 to 126) |
| Leukemia | Allogeneic HSCT | 11.9 (3.1 to 46) |
| ALL | All drug | 2.7 (1.2 to 6.1) |
| AML | High dose chemo | 4.5 (2.9 to 7.1) |
| AML | Low dose chemo | 6.0 (4.7 to 7.7) |
| CLL | Cyclophos | 33.1 (6.9 to 158) |
| CLL | Bendamustin | 43.0 (9.8 to 189) |
| CLL | Ibrutinib | 20.7 (5.9 to 72) |
| CLL | Venetoclax | 39.6 (9.4 to 168) |
| CLL | All drugs | 16.9 (10 to 27) |
| CML | Imatinib | 38.3 (5.4 to 274) |
| CML | 2nd gen TKI | 78.2 (11 to 569) |
| CML | All drug | 31.8 (14 to 71) |
| Other leukemias | All drugs | 12.8 (9.3 to 18) |

*Cells are masked, so it is not possible to identify or back-calculate numbers less than 5.
Abbreviations: ABVD, adriamycin+bleomycin+vinblastine+dacarbazine; ALL, acute lymphatic leukemia; AML, acute myeloid leukemia; BEACOPP, bleomycin+etoposide+adriamycin+cyclophosphamide+vincristine+procarbazine+prednisone; CDK4/6i, Cyclin-dependent kinase 4/6 inhibitors; chemo, chemotherapy; CHOP/CHOEP, cyclophosphamide+hydroxydaunorubicin+vincristine+(etoposide)+prednisone ; CLL, chronic lymphatic leukemia; CML, chronic myeloid leukemia; cyclophos, cyclophosphamide; dabra/tram, dabrafenib/trametinib; enco/bini, encorafenib/binimetinib; ex, excision; gen, generation; HIPEC, hyperthermic intraperitoneal chemotherapy; HR, hazard ratio; HSCT, hematopoietic stem-cell transplantation; ICI, immune checkpoint inhibitors; PARPi, poly(ADP-ribose) polymerase inhibitors; res, resection; TKI, tyrosine kinase inhibitors. TURB, transurethral resection of bladder.
NB: patients could be included in multiple treatment groups.
